# Switching of hypertrophic signalling towards enhanced cardiomyocyte identity and maturity by a GATA4-targeted compound

**DOI:** 10.1186/s13287-023-03623-x

**Published:** 2024-01-02

**Authors:** Lotta Pohjolainen, Sini M. Kinnunen, Samuli Auno, Alexandros Kiriazis, Saana Pohjavaara, Julia Kari-Koskinen, Matej Zore, Mikael Jumppanen, Jari Yli-Kauhaluoma, Virpi Talman, Heikki Ruskoaho, Mika J. Välimäki

**Affiliations:** 1 Drug Research Program and Division of Pharmacology and Pharmacotherapy, Faculty of Pharmacy, University of Helsinki, P.O. Box 56, 00014 Helsinki, Finland; 2 Drug Research Program and Division of Pharmaceutical Chemistry and Technology, Faculty of Pharmacy, University of Helsinki, Helsinki, Finland

**Keywords:** Cardiomyocytes, Maturation, Hypertrophy, GATA4, Isoxazole derivatives

## Abstract

**Background:**

The prevalence of heart failure is constantly increasing, and the prognosis of patients remains poor. New treatment strategies to preserve cardiac function and limit cardiac hypertrophy are therefore urgently needed. Human induced pluripotent stem cell-derived cardiomyocytes (hiPSC-CMs) are increasingly used as an experimental platform for cardiac in vitro studies. However, in contrast to adult cardiomyocytes, hiPSC-CMs display immature morphology, contractility, gene expression and metabolism and hence express a naive phenotype that resembles more of a foetal cardiomyocyte.

**Methods:**

A library of 14 novel compounds was synthesized in-house and screened for GATA4-NKX2-5 reporter activity and cellular toxicity. The most potent compound, 3i-1262, along with previously reported GATA4-acting compounds, were selected to investigate their effects on hypertrophy induced by endothelin-1 or mechanical stretch. Morphological changes and protein expression were characterized using immunofluorescence staining and high-content analysis. Changes in gene expression were studied using qPCR and RNA sequencing.

**Results:**

The prototype compound 3i-1262 inhibited GATA4-NKX2-5 synergy in a luciferase reporter assay. Additionally, the isoxazole compound 3i-1262 inhibited the hypertrophy biomarker B-type natriuretic peptide (BNP) by reducing BNP promoter activity and proBNP expression in neonatal rat ventricular myocytes and hiPSC-CMs, respectively. Treatment with 3i-1262 increased metabolic activity and cardiac troponin T expression in hiPSC-CMs without affecting GATA4 protein levels. RNA sequencing analysis revealed that 3i-1262 induces gene expression related to metabolic activity and cell cycle exit, indicating a change in the identity and maturity status of hiPSC-CMs. The biological processes that were enriched in upregulated genes in response to 3i-1262 were downregulated in response to mechanical stretch, and conversely, the downregulated processes in response to 3i-1262 were upregulated in response to mechanical stretch.

**Conclusions:**

There is currently a lack of systematic understanding of the molecular modulation and control of hiPSC-CM maturation. In this study, we demonstrated that the GATA4-interfering compound 3i-1262 reorganizes the cardiac transcription factor network and converts hypertrophic signalling towards enhanced cardiomyocyte identity and maturity. This conceptually unique approach provides a novel structural scaffold for further development as a modality to promote cardiomyocyte specification and maturity.

**Supplementary Information:**

The online version contains supplementary material available at 10.1186/s13287-023-03623-x.

## Background

Due to the significant increase in prevalence and poor prognosis of cardiovascular diseases, particularly heart failure, new treatments and research concepts aiming to preserve cardiac contractile function and limit cardiac hypertrophy are urgently needed [1]. Human induced pluripotent stem cell-derived cardiomyocytes (hiPSC-CMs) represent a valuable research tool in disease modelling and translational research for e.g., cardiac hypertrophy, arrhythmias and channelopathies [2]. In contrast to adult cardiomyocytes, hiPSC-CMs largely express an immature phenotype in terms of cell size and morphology, contractility, gene expression and metabolic activity [3]. Therefore, several strategies have been employed to advance hiPSC-CM maturation towards a more authentic phenotype to be more suitable for disease modelling and stem cell therapies. Previous reports describe hiPSC-CM maturation as a dynamic process that can be controlled by activation of various physiological and chemical cues, including long-term culture [4], fatty acid exposure [5], electrical stimulation [6], and mechanical stress [7]. Due to complementary and parallel signalling networks, any individual approach alone may not be sufficient for comprehensive structural and functional maturation. Previous approaches studying the effects of chemical and metabolic interventions on cardiomyocyte plasticity and maturation exemplify and provide a viable opportunity for hiPSC-CM maturation. In particular, activation of nuclear receptors by thyroid hormone and/or glucocorticoids has been shown to play a decisive role in normal heart development and cardiomyocyte maturation in vitro and in vivo [8–10]. Additionally, a recent finding suggests that stem cell differentiation into adult cardiomyocytes is stimulated and further promoted by the natural steroidal alkaloid tomaditine [11].

The transcription factors (TFs) GATA1-6 are highly conserved double zinc-finger proteins that, together with other blood- and cardiac-specific regulators, determine the specification of haematopoietic and cardiac cells [12]. Concentration- and context-dependent GATA4 associates with both cardiogenic and hypertrophic signalling cascades in a unique manner, e.g., mitogen-activated protein kinase (MAPK) phosphorylation is required for GATA4-dependent cardiac myocyte survival and hypertrophy but is entirely dispensable for GATA4-induced cardiogenesis [13]. Moreover, several studies have demonstrated that GATA4 is a critical factor for cardiomyocyte differentiation, cardiomyocyte reprogramming, and inherited hypertrophic cardiomyopathies [14–17]. Our previous results showed that activation of GATA4 and NKX2-5 is essential for mechanical stretch-induced cardiomyocyte hypertrophy [18]. Initial activation of GATA4 is followed by upregulation of B-type natriuretic peptide (BNP) and reactivation of foetal genes such as atrial natriuretic peptide (ANP), β-myosin heavy chain, and skeletal muscle α-actin [19]. Substantial data show that cardiomyocyte identity is governed by a limited number of TFs and their interactions with DNA promoters and enhancers. Heterotypic TF interactions that are critical for the direct reprogramming of fibroblasts to cardiomyocytes, including GATA4, MEF2C, TBX5 and HAND2, tend to activate cardiac lineage-specific gene patterns [16, 20, 21]. Additionally, in vivo results demonstrate that GATA4 overexpression has the potential to preserve cardiac function after injury through increased angiogenesis and reduced fibrosis, apoptosis, and drug-induced cardiotoxicity [22–25]. Likewise, GATA4 gene transfer into the heart markedly rescued the loss of regenerative capacity after cryoinjury [14].

We have previously identified GATA4-acting compounds that inhibit the GATA4-NKX2-5 interaction and cause downstream effects on cardiac gene expression, such as BNP and myosin light chain 2 [26–28]. We have further reported that in rodent models by modulating GATA4-NKX2-5 synergy, we were able to reduce the hypertrophic growth of cardiac myocytes [26] and enhance myocardial repair after myocardial infarction and other cardiac injuries [29, 30]. As the modulation of hiPSC-CM identity and maturity is currently a topic of great interest and novel cardiomyocyte-specific therapeutic strategies are warranted, we screened a novel, structurally consistent set of GATA4-acting compounds and explored their potential to inhibit hypertrophic signalling and modulate hiPSC-CM maturation. In particular, the compound candidates were evaluated and ranked according to their effects on GATA4-NKX2-5 synergy and hypertrophic gene expression in endothelin-1 (ET-1) and mechanical stretch models of hypertrophy in hiPSC-CMs. Finally, we utilised molecular pathway analyses to reveal hiPSC-CM maturation-associated processes involving metabolic activity, steroid biosynthesis, fatty acid metabolism and cell cycle exit, implying a change in the maturation status of hiPSC-CMs.

## Methods

### Compounds and reagents

The compound candidates were synthesized at the Division of Pharmaceutical Chemistry and Technology, Faculty of Pharmacy, University of Helsinki (Finland). The syntheses of compounds 3i-1249, 3i-1250, 3i-1251, 3i-1252, 3i-1253, 3i-1254, 3i-1255, 3i-1256, 3i-1258, 3i-1259, 3i-1260, 3i-1261, 3i-1262, 3i-1263 are described in Additional file 1: Methods. The syntheses of compounds 3i-1000, 3i-1022, 3i-1047, 3i-1157, and 3i-1183 have been described previously [26, 27].

### Luciferase screening assays

The primary luciferase screening assays for GATA4-NKX2-5 synergy as well as GATA4 and NKX2-5 reporter activity have been described previously [26, 29, 31]. Briefly, COS-1 cells were plated on a 96-well plate and transfected the next day with three high-activity NKX2-5 binding site-containing luciferase promoter (p3HA-luc), GATA4 and NKX2-5 expression plasmids (pMT2-GATA4 and pMT-NKX2-5, respectively) using FuGENE 6 reagent (Promega). After 6 h, the cells were treated with compounds or 0.1% DMSO in Dulbecco’s Modified Eagle’s Medium (DMEM) with 10% foetal bovine serum (FBS), 100 U/mL penicillin, and 100 µg/mL streptomycin. The luciferase signal was measured after 24 h by using Neolite Reporter Gene Assay System (PerkinElmer, Turku, Finland) and Victor2 plate reader (PerkinElmer). In GATA4 reporter activity assay, the cells were transfected with GATA4 binding site containing luciferase promoter (NP-112), and in NKX2-5 assay, with p3HA-luc, in addition to the relevant expression plasmids described above.

### AlphaScreen

The AlphaScreen (Amplified Luminescent Proximity Homogenous Assay) was used to measure the effects of compound 3i-1262 on GATA4-NKX2-5 interaction. Detailed description is provided in Additional file 1: Methods.

### Neonatal rat ventricular cardiomyocyte culture and BNP reporter assay

Neonatal rat ventricular myocytes (NRVMs) were prepared from 1- to 4-day-old Wistar rats as described previously [26]. The rats were euthanised by decapitation. The cells were seeded on a gelatin-coated 96-well plate at 30,000–50,000 cells/well and cultured in DMEM/F12 culture medium containing 2.5 mM L-glutamine, supplemented with 10% FBS and 100 U/mL penicillin, and 100 µg/mL streptomycin. On the next day, the media was changed to complete serum-free media (CSFM; DMEM/F12, 2.5 mg/mL bovine serum albumin, 1 μM insulin, 2.5 mM L-glutamine, 32 nM selenium, 2.8 mM sodium pyruvate, 5.64 μg/mL transferrin, 1 nM T3, 100 U/mL penicillin, and 100 µg/mL streptomycin) and cells were transfected with p(∆-534 bp/ + 4 bp)BNP-luciferase (-534rBNP) reporter vector at 100 ng/well using FuGENE 6 reagent 3:1 ratio to DNA [32]. After a 24-h transfection, the media was changed and 80 µL of medium containing the compounds or vehicle 0.1% DMSO was added. After 1 h, 20 µL of ET-1 or plain medium was added, final concentrations being 10 µM or 30 µM for compounds and 100 nM for ET-1. Luciferase activity was measured after 24 h using Neolite Reporter Gene Assay System (PerkinElmer).

### Human pluripotent stem cells and human pluripotent stem cell-derived cardiomyocytes

The human iPS(IMR90)-4 line derived from a female donor with lentiviral reprogramming was purchased from WiCell (Madison, WI, USA). The human induced pluripotent stem cells (hiPSCs) were maintained in Essential 8 medium on Matrigel-coated 6-well plates and passaged 1:15 approximately every four days using Versene. When the cultures were 80–95% confluent, differentiation was initiated using the previously described differentiation protocol [33–35]. After differentiation and a 4-day metabolic selection through glucose deprivation, hiPSC-CMs (> 90% pure cultures) were seeded in RB+ medium supplemented with 10% FBS on flexible collagen I-coated 6-well BioFlex® culture plates (Flexcell International Corporation, Hillsborough, NC, USA) with additional Matrigel coating at a density of 700,000–800,000 cells/well for mechanical stretching or on gelatin-coated 96-well plates at density of 10,000–20,000 cells/well for high-content analysis (HCA) and for cell viability assay. The hiPSC-CMs were allowed to attach for 48 h, after which the medium was changed to serum-free RB+. Before conducting the hypertrophy experiments, cultures were maintained by changing fresh RB+ approximately every four days until day 25–43 from the start of differentiation. For cell viability assay, hiPSCs were plated on gelatin-coated 96-well plates at 10,000 cells/well and incubated overnight before adding the compounds.

### Cell viability assay

The cell viability was assessed by measuring mitochondrial metabolic activity with 3-(4,5-dimethyl-2-thiazolyl)-2,5-diphenyltetrazolium bromide (MTT) assay, as described previously [36]. Briefly, MTT was added to the cells at a final concentration of 0.5 mg/mL. After a 2-h incubation at 37 °C in a humidified atmosphere of 5% CO_2_, the medium was removed, and the formazan crystals were dissolved in 200 μL of DMSO per well. Absorbance was measured with Bio-Rad plate reader (Hercules, CA, USA) at 550 nm subtracting the absorbance at 650 nm as background. To calculate the cell viability, the absorbance values were compared to the absorbance of untreated cells. In each independent experiment, three technical replicates were used for each treatment and the average of the technical replicates was used for analysis as *N* = 1.

### Cyclic mechanical stretching

On day 29–43 after differentiation initiation, hiPSC-CMs on Bioflex culture plates were mechanically stretched for 24 h, 48 h and 72 h using Flexcell FX-5000 Tension System (Flexcell International Corporation) [37]. The cyclic stretch varied between 10 and 21% elongation at frequency of 0.5 Hz. Unstretched control cells from the same differentiation were maintained on similar Bioflex culture plates and in the same environmental conditions, but no stretch was applied.

### Immunofluorescence staining and high-content analysis

For HCA, the hiPSC-CMs on 96-well plates were exposed to the compounds and ET-1 or vehicle for 24 h or 96 h on day 25–34 after the initiation of the differentiation. 5-Bromo-2′-deoxyuridin (BrdU; Abcam) was added to the cells to be analysed for proliferation 24 h before fixation at a concentration of 10 µM. Brefeldin A (Invitrogen) was added to the cells to be stained for pro-B-type natriuretic peptide (proBNP) 3 h before fixation to block exocytosis of proBNP-containing vesicles. The cells were fixed and stained for cardiac troponin T (cTnT), proBNP, BrdU, GATA4 and 4′,6-diamidino-2-phenylindole (DAPI) as described in Additional file 1: Methods.

The CellInsight™ CX5 High Content Screening Platform (Thermo Scientific) was used to image and analyse the cells. Images were collected using a 10 × objective (Olympus UPlanFL N 10 × /0.3) with 16–25 sites per well to examine more than 100 cells in each well. The images were analysed with the Cellomics software (Thermo Scientific) using the Cellomics Compartmental Analysis BioApplication. First, the nuclei were identified based on DAPI staining. To analyse only cardiomyocytes, the average intensity of cTnT staining within the nuclei was quantified and only cells expressing cTnT above the set threshold were selected for further analysis. Then the average intensity of BrdU staining was quantified from nuclear area and the average intensity of proBNP staining was quantified from the perinuclear region defined as an 8-pixel ring around each nucleus as described previously [30]. Cells were classified into BrdU and proBNP positive and negative cells based on their intensity. The reference level was set individually for each experiment. In addition, nuclear DAPI and nuclear and perinuclear GATA4 and cTnT staining intensities were quantified. Analysis of cell cycle was performed by measuring the nuclear DNA content of individual cells. The threshold for high DNA cells representing cells in the G2 phase of the cell cycle was set manually in each experiment using DNA content frequency histograms to adjust for minor variation in staining intensity. The lower threshold for “high DNA cells” was set in the middle of G1 and G2 peaks. Each experiment included at least two technical replicates, i.e., two parallel wells of each treatment group. The average of technical replicates, representing *N* = 1, was used in the analysis.

### Analysis of RNA

The total RNA was isolated using NucleoSpin RNA kit (Macherey–Nagel, Düren, Germany) according to the manufacturer’s instructions. RNA concentration and quality were analysed with a NanoDrop 1000 spectrophotometer (Thermo Fisher Scientific, Waltham, MA, USA) for qPCR and with 4200 TapeStation (Agilent, Santa Clara, CA, USA) for RNA sequencing (RNAseq). The RNA expression was analysed by qPCR and RNAseq as described in Additional file 1: Methods. The RNAseq results regarding the stretch response without 3i-1262 have been published previously [37].

### Functional analysis of differentially expressed genes

Gene Ontology (GO) enrichment analyses were performed using the GOrilla tool (version updated 6th of March 2021, available at http://cbl-gorilla.cs.technion.ac.il/). Differentially expressed genes (FC > 1.5, *p* < 0.01) were analysed against a background set consisting of all the genes expressed in our samples, defined as genes with a detected signal in at least two samples in one treatment group. For running the analysis, threshold of *p* < 0.0001 was used. False discovery rate (FDR)-adjusted *p*-values were calculated after the analysis and an FDR-adjusted *p*-value < 0.05 was considered significant. KEGG pathway analysis was performed using WebGestalt (version 2019, available at http://www.webgestalt.org/). For multiple testing, the Benjamini–Hochberg method was used and *p* < 0.05 was considered significant.

### Statistics

Results are expressed as mean ± standard error of the mean (SEM). Statistical analysis of RNAseq results was performed as a pairwise comparison using a Wald test in DESeq2 software. Genes, for which FC > 1.5 and Benjamini–Hochberg adjusted *p* < 0.01, were defined as differentially expressed. All other statistical analyses were performed in IBM SPSS Statistics 25 software. Statistical significance was evaluated by one-way analysis of variance (ANOVA) followed by a Tukey post-hoc test. In case of unequal variances, Welch ANOVA was used followed by Games-Howell. Independent samples t-test, paired samples t-test or Mann–Whitney U test was used to determine the statistical difference between two groups. A *p*-value of < 0.05 was considered statistically significant.

## Results

### Inhibition of GATA4-NKX2-5 interaction

**Fig. 1:**
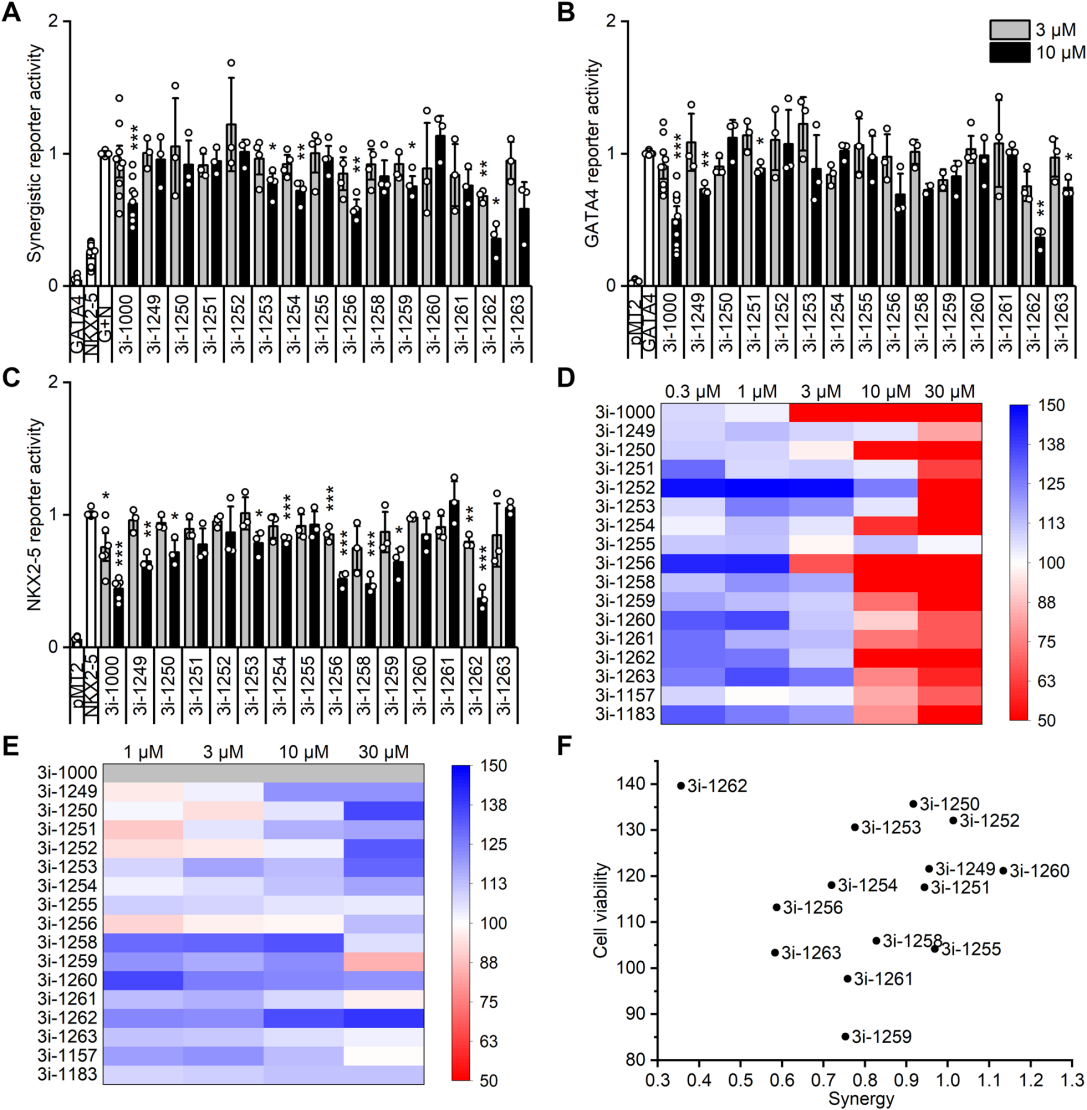
Activity of the novel compounds and toxicity screening. The activity of the compounds was tested in a luciferase reporter assay on **A** GATA4-NKX2-5 synergy, **B** GATA4 activity and **C** NKX2-5 activity in COS-1 cells. The toxicity of the compounds to **D** hiPSCs and **E** hiPSC-CMs was studied using the MTT assay. Results are expressed as mean ± SEM (*N* = 3–10, except 2 for B, 3i-1258 10 µM and 3i-1259 3 µM). *p < 0.05, ** *p* < 0.01, ***p < 0.001 vs. control group (**A**, G + N; **B**, GATA4; **C**, NKX2-5) within compounds (Levene’s test followed by independent samples t-test). **F** Compilation chart of novel GATA4-acting compounds plotted as a function of GATA4-NKX2-5 synergy (10 µM) and hiPSC-CM viability (30 µM)

We synthesised and screened a structurally focused library of 14 novel compounds for GATA4-NKX2-5 interaction in the luciferase assay, where GATA4 and NKX2-5 synergistically activate luciferase reporter expression in a vector containing three artificial NKX2-5 binding sites (NKE) (Fig. 1A) [26, 27]. Several compounds (3i-1253, 3i-1254, 3i-1256, 3i-1259 and 3i-1262) inhibited the synergy of GATA4 and NKX2-5 in a concentration-dependent manner. However, only one of the novel compounds, 3i-1262, was a more potent inhibitor (64% vs. 38%) than our previous lead compound 3i-1000. In addition to the synergy assay, we studied the effects of the compounds on GATA4 reporter activity using a tandem GATA site-containing luciferase reporter (Fig. 1B) and on NKX2-5 reporter activity using the three NKE site-containing vector (Fig. 1C). Compounds 3i-1000, 3i-1249 and 3i-1262 inhibited both GATA4 and NKX2-5 reporter activity in a concentration-dependent manner, and several other compounds showed a significant inhibition of NKX2-5 reporter or GATA4 reporter activity. To further confirm the effect of compound 3i-1262 on the GATA4-NKX2-5 interaction, we used an AlphaScreen assay that demonstrated concentration-dependent inhibition of the GATA4-NKX2-5 interaction (Additional file 1: Fig. S1). Furthermore, we explored the possible off-targets by screening the activity of compound 3i-1262 against the commercial kinase library and did not identify any obvious activity among the kinase proteins (Additional file 1: Table S1).

### Compound 3i-1262 is nontoxic to cardiomyocytes

We studied the toxicity of the compounds in both hiPSCs and hiPSC-CMs by exposing the cells to the compounds for 24 h and measuring viability with the MTT assay (Fig. 1D–E). As reported previously, stem cells are more sensitive to compound toxicity than cardiomyocytes in general [34]. Here, the tentative structure–toxicity relationship for stem cell viability was drafted (Additional file 1: Fig. S2). Only one compound, 3i-1255, was nontoxic to both cell types in the range of 0.3–30 µM. The most potent inhibitor of GATA4-NKX2-5 synergy, compound 3i-1262, decreased the viability of hiPSCs at high concentrations but was nontoxic to cardiomyocytes even at 30 µM. In contrast, 3i-1262 and several other compounds appeared to increase the mitochondrial metabolic activity of hiPSC-CMs as measured by the MTT assay (Fig. 1E). Based on the primary screening and toxicity measurements, the most potent compound 3i-1262 (Fig. 1F and Additional file 1: Table S2) was selected for further characterisation and compared thoroughly to the other previously reported GATA4-acting compounds 3i-1000, 3i-1022, 3i-1047, 3i-1157 and 3i-1183 [27].

### Inhibition of BNP activation and gene expression

**Fig. 2:**
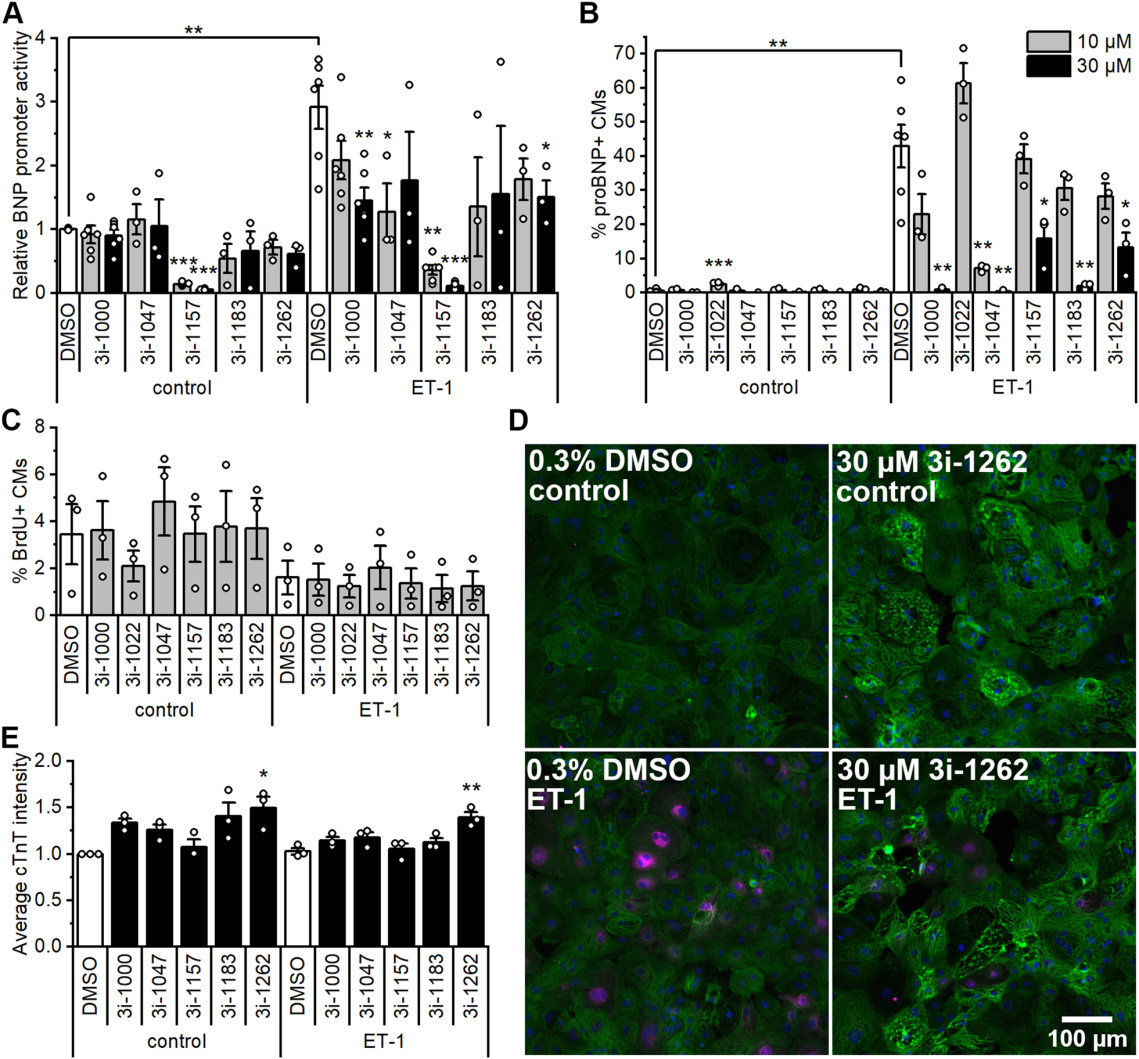
Effects of compounds and endothelin-1 (ET-1) on B-type natriuretic peptide (BNP) promoter activity, pro-B-type natriuretic peptide (proBNP) expression, cell proliferation, and cardiac troponin T (cTnT) expression. **A** BNP promoter activity was studied in NRVMs by transfecting the cells with luciferase reporter vector containing -534 bp of BNP promoter for 24 h followed by a 24 h exposure to 3i-compounds and ET-1 at 100 nM. Luciferase activity was normalised to the DMSO control. **B–E** hiPSC-CMs were exposed to 3i-compounds, ET-1 at 100 nM and 5-bromo-2'-deoxyuridine (BrdU) at 10 µM for 24 h, after which hiPSC-CMs were fixed and stained for DNA, cTnT, proBNP and BrdU. **B** Percentage of proBNP+ cardiomyocytes based on the average intensity of proBNP staining in the perinuclear region of cTnT+ cells (cardiomyocytes). **C** The percentage of BrdU+ cells based on the average intensity of BrdU staining in the nuclei of cTnT+ cells. **D** Representative images of control hiPSC-CMs (DMSO) and hiPSC-CMs treated with 30 µM 3i-1262 with and without ET-1. Cells were stained for DAPI (blue), cTnT (green), and proBNP (magenta). **E** Average intensity of cTnT staining was measured within the nuclear area and normalised to the DMSO control. The results are expressed as the mean ± SEM (*N* = 3–6). *p < 0.05, **p < 0.01, ***p < 0.001 vs. DMSO within the control or ET-1 group (one-way ANOVA followed by Tukey or Welch ANOVA followed by Games-Howell) or DMSO control vs. DMSO ET-1 (independent samples t-test)

BNP expression is a well-established biomarker of cardiomyocyte hypertrophy that increases in response to cardiac stress and injury [38]. We studied the effects of compound 3i-1262 and previously reported GATA4-acting compounds [27] on BNP gene activation in NRVMs. NRVMs were transfected with a luciferase reporter vector containing -534 bp of the BNP promoter for 24 h and then exposed to compounds with and without 100 nM ET-1 for 24 h. Compounds 3i-1000, 3i-1047, 3i-1157 and 3i-1262 significantly attenuated ET-1-induced BNP activation (Fig. 2A), and 3i-1000, 3i-1157 and 3i-1262 showed concentration-dependent responses. Compound 3i-1157 was the most potent inhibitor, and it also decreased baseline BNP activation.

Next, we examined the effects of GATA4-targeting compounds on the hypertrophy of hiPSC-CMs using HCA. The analysis of proBNP expression in hiPSC-CMs revealed that compounds 3i-1000, 3i-1047, 3i-1183, and 3i-1262 attenuated the ET-1-induced hypertrophic response in a concentration-dependent manner, with 3i-1047 being the most potent inhibitor (Fig. 2A–B). Decreased proBNP expression following compound 3i-1262 treatment observed using HCA was supported by Western blotting. Based on proBNP protein levels, the compound 3i-1262 at a 30 µM concentration significantly attenuated the hypertrophic response induced by ET-1 (Additional file 1: Fig. S3). As cardiomyocytes have very limited proliferative capacity and develop hypertrophy instead of proliferation in response to cardiac overload, we also studied hiPSC-CM proliferation by staining and measuring the intensity of BrdU incorporation into DNA. Based on the BrdU assay, none of the compounds had a significant effect on cardiomyocyte proliferation after a 24-h treatment (Fig. 2C). Intriguingly, compound 3i-1262 induced a significant change in cytoskeleton arrangement and cTnT expression in hiPSC-CMs, as seen with cTnT staining (Fig. 2D–E). Quantification of cTnT staining intensities of hiPSC-CMs treated with different compounds compared to the DMSO control is shown in Fig. 2E.

**Fig. 3:**
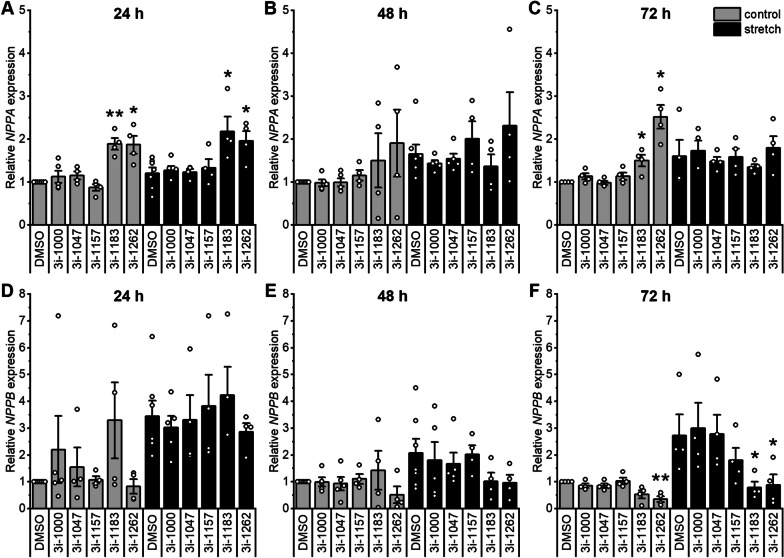
Effects of compounds and mechanical stretch on hypertrophic gene expression in hiPSC-CMs. Relative NPPA **A**–**C** and NPPB **D**–**F** mRNA expression in hiPSC-CMs was measured by qPCR after a 24 h, 48 h, and 72 h exposure to compounds at 30 µM and cyclic mechanical stretch (10–21%, 0.5 Hz). The results are expressed as the mean ± SEM (*N* = 4–7). *p < 0.05, **p < 0.01 vs. DMSO within the control or stretch group (paired samples t-test)

We also explored the effects of compounds on mechanical stretch in hiPSC-CMs by measuring atrial and B-type natriuretic peptide mRNA (*NPPA* and *NPPB*) levels (Fig. 3). Compounds 3i-1183 and 3i-1262 significantly inhibited stretch-induced *NPPB* gene expression after 72 h (Fig. 3F). Compound 3i-1262 also decreased baseline *NPPB* levels after 72 h. On the other hand, 3i-1183 and 3i-1262 increased baseline *NPPA* expression, especially after 24 h and 72 h of exposure (Fig. 3A, C).

### Changes in cardiomyocyte morphology

**Fig. 4:**
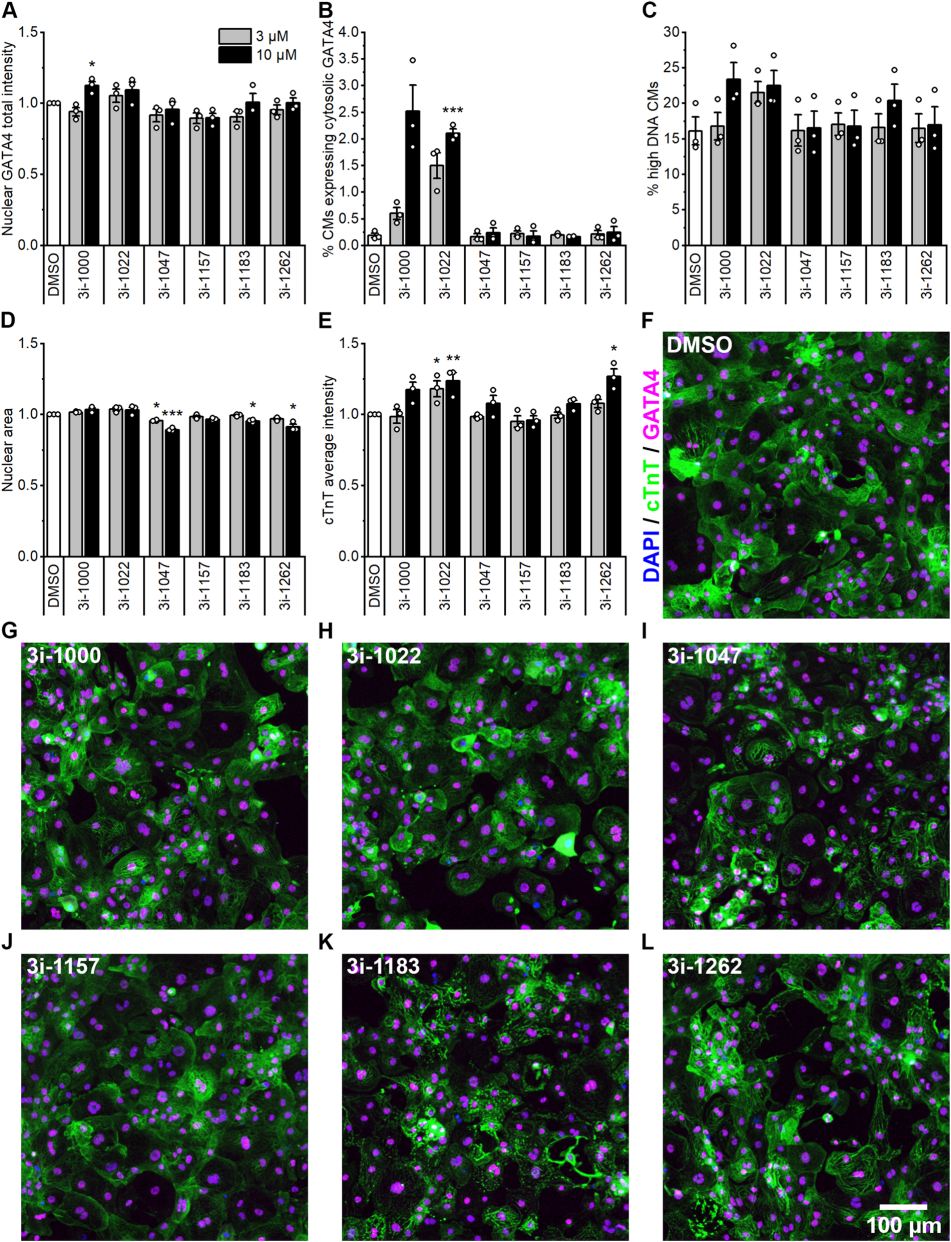
Effects of GATA4-targeted compounds on GATA4 protein expression, DNA content and cardiac troponin T (cTnT) intensity in hiPSC-CMs. After a four-day exposure to compounds, hiPSC-CMs were fixed and stained for DNA, cTnT, and GATA4. **A** Average intensity of GATA4 staining in nuclei. **B** The percentage of cells expressing GATA4 in the perinuclear area is based on the intensity of GATA4 staining in cTnT+ cells. **C** The percentage of cells with high total intensity of DNA staining representing cells in the G2 phase of the cell cycle. **D** Average nuclear area is based on DAPI staining. **E** cTnT intensity was measured in the perinuclear area of the cTnT+ cells. **F**–**L** Representative images of immunofluorescence-stained hiPSC-CMs treated with 0.1% DMSO (**F**) and compounds at 10 µM **G**–**L**, blue = DAPI, green = cTnT, magenta = GATA4. The results are expressed as the mean ± SEM (*N* = 3). *p < 0.05, **p < 0.01, ***p < 0.001 versus DMSO control (All treatment groups at one concentration were compared to DMSO control at the same time using one-way ANOVA followed by the Tukey test or in case of unequal variances Welch ANOVA followed by the Games-Howell test)

We characterised the effects of the compounds on hiPSC-CM morphology and GATA4 expression in a 4-day experiment. Only one compound, 3i-1000 at 30 µM, slightly increased GATA4 staining intensity in nuclei (1.13-fold, *p* = 0.028; Fig. 4A). While GATA4 is classically localised in the nucleus, an atypical increase in GATA4 expression in the cytosol was detected in response to the more toxic compounds 3i-1000 and 3i-1022 at 10 µM [27] (Fig. 4B and Additional file 1: Fig. S4). These two compounds also showed a trend towards increased DNA content, suggesting that more cells entered the G2 phase of the cell cycle (Fig. 4C). The nuclear area of hiPSC-CMs was decreased by compounds 3i-1047, 3i-1183 and 3i-1262 (Fig. 4D). Compounds 3i-1262 and 3i-1022 increased cTnT expression (Fig. 4E), which is in line with the similar observation after a 24-h exposure to 3i-1262 (Fig. 2E). In 3i-1022-treated hiPSC-CMs, increased average cTnT intensity was related to a few considerably high-intensity cells (Fig. 4H), while in 3i-1262-treated cells, cTnT appeared to accumulate in spots in most cells (Fig. 4L). To further investigate the effects of compound 3i-1262 and ET-1 on the expression of α-actinin and cTnT in hiPSC-CMs, Western blotting was performed. The results indicated that 24 h exposure to 3i-1262 did not significantly alter the expression of α-actinin or cTnT, however, compound 3i-1262 diminished the ET-1 induced increase in α-actinin expression (Additional file 1: Fig. S5).

### Compound 3i-1262 induces cardiomyocyte metabolic processes and attenuates cell cycle processes in hiPSC-CMs

We selected compound 3i-1262, the most potent inhibitor of GATA4-NKX2-5 synergy (Fig. 1A, F), for RNAseq analysis. RNAseq was performed in 72-h stretched and unstretched 3i-1262 and DMSO-treated hiPSC-CMs. Overall, principal component analysis showed separation of three groups defined by the first principal component: 3i-1262-treated stretched, 3i-1262-treated unstretched and both DMSO-treated samples (Additional file 1: Fig. S6). This result suggests that 3i-1262 has a pronounced effect on hiPSC-CMs, while the 72-h stretch had a minor effect on hiPSC-CMs, as we previously described [37]. Interestingly, 3i-1262-treated stretched samples resembled DMSO-treated samples more than 3i-1262-treated unstretched samples. The second principal component showed separation of two groups based on individual experiments (Experiments 1 and 4 were separated from Experiments 2 and 3). This result demonstrates that although the cells are from the same origin, there is biological variation between differentiations.

**Fig. 5:**
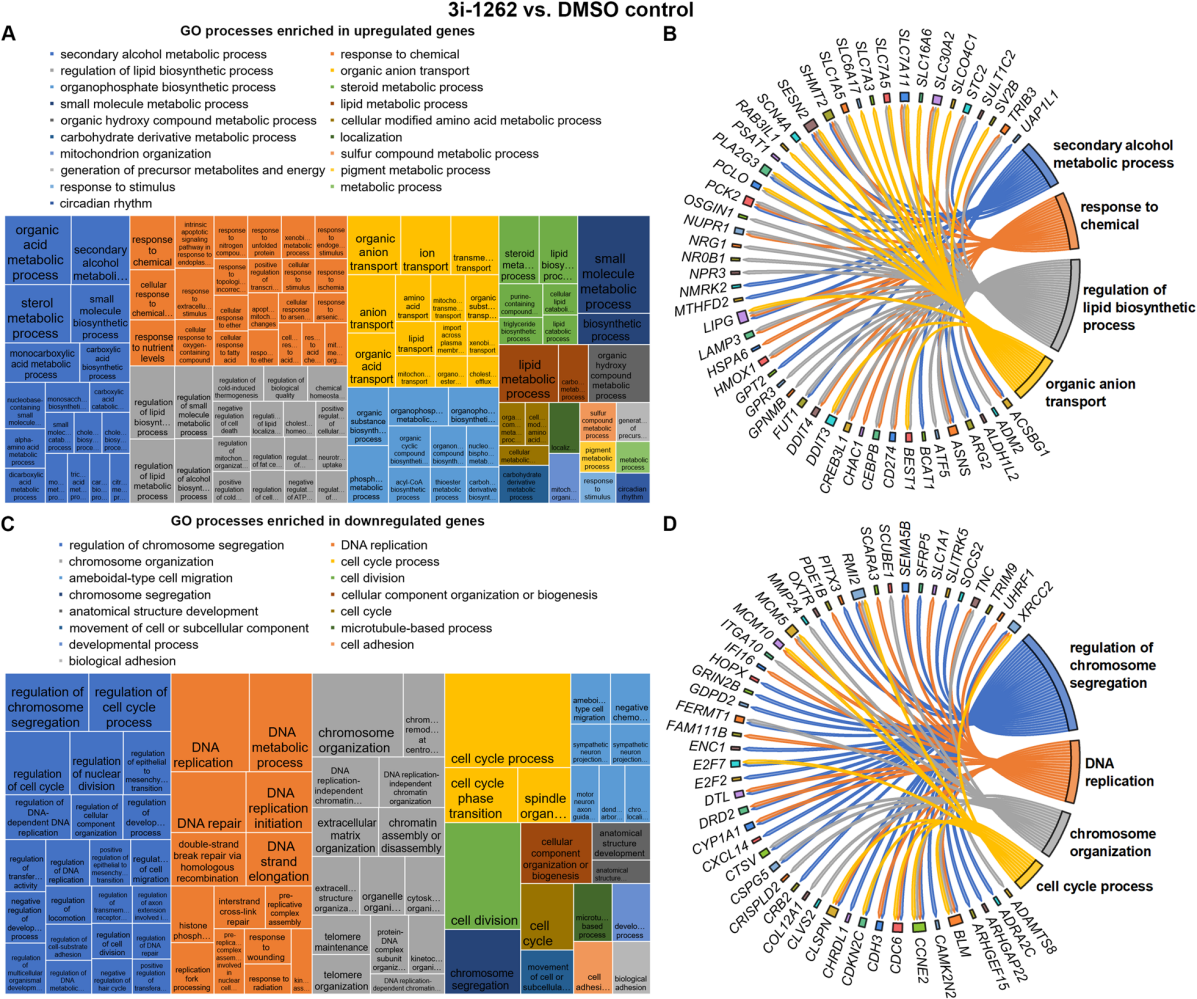
Biological processes enriched in differentially expressed genes in response to 3i-1262 in hiPSC-CMs. **A** Revigo treemap of biological processes enriched in upregulated genes based on gene ontology (GO) analysis. **B** Top 50 upregulated genes associated with the four largest GO categories. **C** Revigo treemap of biological processes enriched in downregulated genes based on GO analysis. **D** Top 50 downregulated genes associated with the four largest GO categories. **A**, **C** The rectangle size in the treemaps reflects the significance of the GO term. Related GO terms are grouped into clusters visualised with different colours

In response to 3i-1262 alone, 1,192 genes were upregulated, and 1,216 genes were downregulated (FC > 1.5, *p* < 0.01). GO enrichment analysis of biological processes revealed that many upregulated genes were associated with metabolic processes (Fig. 5A–B). In particular, secondary alcohol metabolic processes, including sterol metabolism and lipid biosynthetic processes, were enriched in response to 3i-1262. Accordingly, the most significantly enriched pathways with KEGG pathway analysis were biosynthesis of amino acids, steroid biosynthesis, and metabolic pathways (Additional file 1: Table S3). Additionally, the most significantly enriched biological processes included organic anion transport and response to chemicals (Fig. 5A–B). In contrast, downregulated genes in response to 3i-1262 treatment displayed enrichment for biological processes related to the cell cycle and cell division, including chromosome segregation and DNA replication (Fig. 5C–D). Moreover, biological adhesion and cell adhesion were among the enriched downregulated processes. These changes were also supported by KEGG analysis, in which downregulated genes demonstrated enrichment for focal adhesion and ECM-receptor interaction in addition to cell cycle and DNA replication (Additional file 1: Table S4).

### Compound 3i-1262 induces maturation-related gene expression in hiPSC-CMs

Since the increased expression of genes related to metabolic processes and decreased expression of genes associated with the cell cycle indicate cardiomyocyte maturation [3, 39–41], we investigated specific genes related to cardiomyocyte maturation. The maturation of cardiomyocytes occurs on multiple levels, including metabolism, structure, function/electrophysiology, and the cell cycle [3, 39–41]. Some of these features can be experimentally measured at the marker gene level [39–42].

**Fig. 6:**
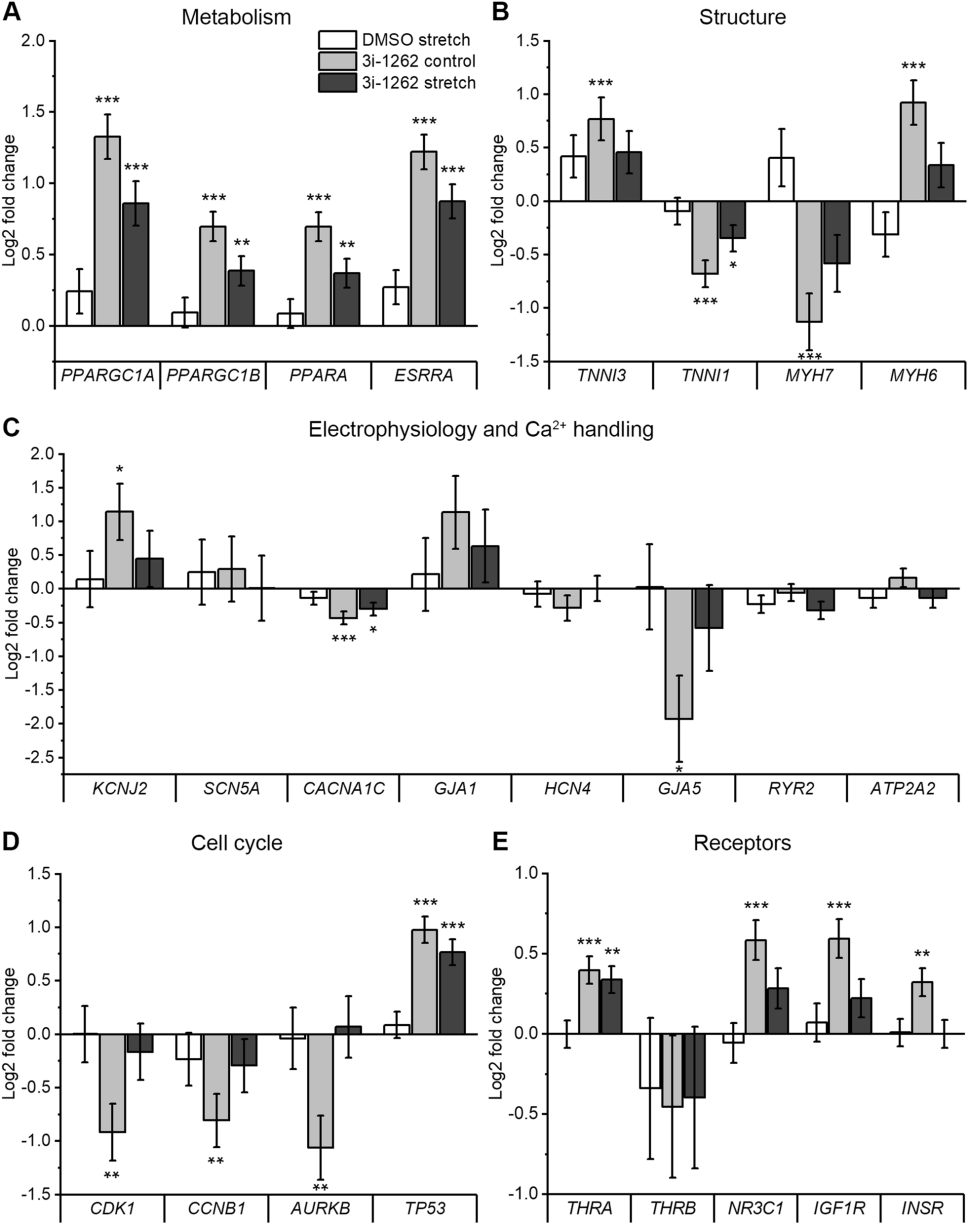
Maturation-related gene expression changes in response to compound 3i-1262. Key genes related to metabolic (**A**), structural (**B**), functional (**C**) and cell cycle (**D**) maturation and genes coding for receptors of key maturation-promoting hormones. hiPSC-CMs were exposed to 3i-1262 at 30 µM and a 72 h stretch. RNA was sequenced, and log2-fold changes (± SEM) relative to the unstretched DMSO control are presented (*N* = 4). *p < 0.05, **p < 0.01, ***p < 0.001 versus DMSO control (Wald test). ATP2A2; ATPase sarcoplasmic/endoplasmic reticulum Ca^2+^ transporting 2; AURKB, aurora kinase B; CACNA1C, calcium voltage-gated channel subunit alpha1 C; CCNB1, G2/mitotic-specific cyclin-B1; CDK1, cyclin-dependent kinase 1; ESRRA, oestrogen related receptor alpha; GJA1, gap junction alpha-1 (Connexin-43); GJA5, gap junction alpha-5 (Connexin-40); HCN4, hyperpolarisation activated cyclic nucleotide gated potassium channel 4; IGF1R, insulin like growth Factor 1 receptor; INSR, insulin receptor; KCNJ2, inward rectifier potassium channel 2; MYH6, myosin heavy chain 6; MYH7, myosin heavy chain 7; NR3C1, nuclear receptor subfamily 3 Group C member 1; PPARA, peroxisome proliferator-activated receptor alpha; PPARGC1A, peroxisome proliferator-activated receptor gamma coactivator 1-alpha; PPARGC1B, Peroxisome proliferator-activated receptor gamma coactivator 1-beta; RYR2, ryanodine receptor 2; SCN5A, sodium voltage-gated channel alpha subunit 5; THRA, thyroid hormone receptor alpha; THRB, thyroid hormone receptor beta; TNNI1, troponin I1, slow skeletal type; TNNI3, troponin I3, cardiac type; TP53, tumour protein P53

Key features of metabolic maturation include a shift from glycolytic to oxidative metabolism, increased fatty acid β-oxidation, and increased mitochondrial number and function [3, 39–41]. Multiple studies suggest that peroxisome proliferator-activated receptor gamma coactivator 1*α* (PGC-1*α*) and/or 1β (PGC-1β) are key regulators of the metabolic maturation processes in both developing hearts (activation) and hypertrophied hearts (deactivation) [3, 39–46]. At the gene level, the increased expression of the genes coding for PGC-1α and PGC-1β (*PPARGC1A* and *PPARGC1B*, respectively) as well as their main interaction partners peroxisome proliferator-activated receptor alpha (*PPARA*) and oestrogen-related receptor alpha (*ESRRA*), is observed upon maturation [39, 40, 47]. Interestingly, all these genes were upregulated in response to 3i-1262 (Fig. 6A).

In addition to enhanced energy metabolism, structural maturation of cardiomyocytes is required for increased contractile force production [3, 39, 40]. The structural transcriptomic changes include not only increased expression of mature sarcomere components but also contractile protein isoform switching [39]. During cardiac development, slow skeletal troponin I (ssTnI) is replaced by cardiac troponin I (cTnI), and the myosin heavy chain (MHC) switches from isoform α to β [3, 39, 48]. Here, we observed a shift in troponin I gene expression in response to 3i-1262: foetal-type troponin I (*TNNI1*) decreased, while adult-type troponin I (*TNNI3*) increased, although *TNNI1* was still the predominant type (Fig. 6B). On the other hand, the expression of MHC-β (*MYH7*) decreased, while MHC-*α* (*MYH6*) expression increased. Of note, *MYH7* is expressed more highly than *MYH6* in hiPSC-CMs at baseline (DMSO control) (Additional file 2: Table S5).

Functionally mature cardiomyocytes have different electrophysiological and calcium-handling properties compared to immature cardiomyocytes [39, 40]. During maturation, the action potentials of cardiomyocytes change: cardiomyocytes lose their automaticity, obtain a more negative resting membrane potential, and increase action potential duration and amplitude. As a cue for electrophysiological maturation, the expression of genes encoding channel proteins and gap junction proteins changes. Key transcriptomic alterations of electrophysical maturation include increased expression of the inward rectifier potassium channel 2 coding gene *KCNJ2,* sodium channel protein type 5 subunit alpha coding gene *SCN5A*, voltage-dependent L-type calcium channel subunit alpha-1C coding gene *CACNA1C* and gap junction protein alpha 1 coding gene *GJA1* and decreased expression of the potassium/sodium hyperpolarisation-activated cyclic nucleotide-gated channel 4 coding gene *HCN4* and gap junction protein alpha 5, *GJA5* [39, 40, 49]. Of these genes, 3i-1262 induced upregulation of *KCNJ2* and a switch from *GJA5* to *GJA1* (Fig. 6C). In contrast, *CACNA1C* was downregulated in response to 3i-1262, while no significant increase in *SCN5A* or decrease in *HCN4* was observed in response to 3i-1262 or stretch. Although hiPSC-CMs express calcium-handling proteins, the levels of expression might differ from those in mature cardiomyocytes [39, 40]. In particular, increased expression of ryanodine receptor 2 coding gene (*RYR2*) and ATPase sarcoplasmic/endoplasmic reticulum Ca^2+^ transporting 2 coding gene (*ATP2A2*, also known as *SERCA2*) is linked to cardiomyocyte maturation, yet we did not find any significant changes in the expression of these genes (Fig. 6C).

During cardiomyocyte maturation, cell cycle activity is altered, resulting in a loss of cell proliferation capacity [3]. At the level of gene expression, multiple key cell cycle regulators are downregulated, particularly genes coding for cyclin-dependent kinase 1 (*CDK1*), cyclin B1 (*CCNB1*) and aurora kinase B (*AURKB*) [39]. Remarkably, all these genes were downregulated in 3i-1262-treated hiPSC-CMs (Fig. 6D). Moreover, in agreement with the finding that cell cycle arrest is driven by increased expression of *TP53* (p53) resulting from inhibition of mTOR [41], the expression of *TP53* was markedly increased in response to 3i-1262 (Fig. 6D). To confirm the results gained from RNA sequencing, we selected five maturation-related genes for validation by qRT-PCR in hiPSC-CMs: *PPARGC1A*, *CCNB1*, *MYH6*, *MYH7*, and *AURKB*. Overall, we noted consistent findings using both qRT-PCR and RNAseq analyses (Additional file 1: Fig. S7).

Furthermore, we noted an increase in the expression of receptors for triiodothyronine (T3), glucocorticoids, and insulin-like growth factors (IGFs), which are known to promote cardiomyocyte maturation. More specifically, thyroid hormone receptor alpha coding gene (*THRA*) (but not thyroid hormone receptor beta (*THRB*)), glucocorticoid receptor coding gene nuclear receptor subfamily 3 Group c member 1 (*NR3C1*), IGF receptor coding genes insulin-like growth Factor 1 receptor (*IGF1R*) and insulin receptor (*INSR*) were upregulated (Fig. 6E), suggesting that pathways related to these maturation-promoting hormones are activated.

Overall, the review of individual maturation-related genes confirms the results of GO enrichment analysis. Compound 3i-1262 induced gene expression changes related to metabolic and cell cycle maturation, while an equally strong pattern for cardiomyocyte maturation was not observed in genes related to structural and functional maturation.

### Mechanical stretch counteracts the transcriptomic changes induced by compound 3i-1262 in hiPSC-CMs

**Fig. 7:**
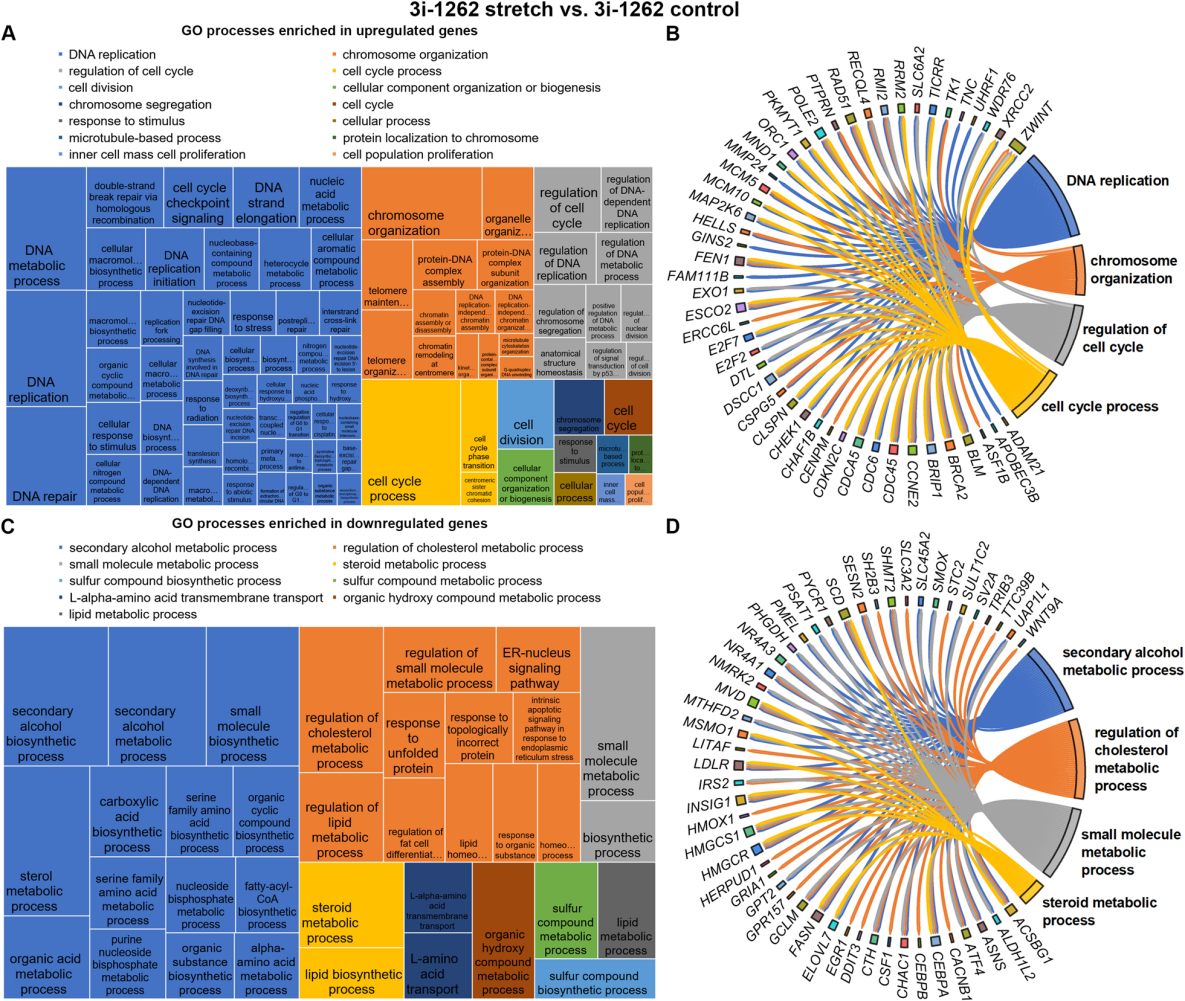
Biological processes enriched in differentially expressed genes in response to stretch in 3i-1262-treated hiPSC-CMs. **A** Revigo treemap of biological processes enriched in upregulated genes based on gene ontology (GO) analysis. **B** Top 50 upregulated genes associated with the four largest GO categories. **C** Revigo treemap of biological processes enriched in downregulated genes based on GO analysis. **D** Top 50 downregulated genes associated with the four largest GO categories. **A**, **C** The rectangle size in treemaps reflects the significance of the GO term. Related GO terms are grouped into clusters visualised with different colours

Finally, we investigated the transcriptomic changes in response to cyclic mechanical stretch in 3i-1262-treated hiPSC-CMs. We found that in response to stretch, 260 genes were upregulated, and 197 genes were downregulated (FC > 1.5, *p* < 0.01). Interestingly, based on GO enrichment analysis of biological processes, the upregulated genes showed enrichment for several cell cycle- and DNA replication-related processes, while the downregulated genes displayed enrichment for metabolic processes (Fig. 7). Hence, the same processes that were enriched in upregulated genes in response to 3i-1262 were downregulated in response to stretch, and correspondingly, the downregulated processes in response to 3i-1262 were upregulated in response to stretch (Additional file 1: Fig. S8). As a result, mechanical stretching reversed the effect of compound 3i-1262. In fact, when we compared the differentially expressed genes, significant overlap between the gene sets was observed: 239 of the 260 upregulated genes in response to stretch were downregulated in response to 3i-1262, and 180 of the 197 downregulated genes in response to stretch were upregulated in response to 3i-1262 (Additional file 1: Fig. S8). However, it is noteworthy that the number of differentially expressed genes was markedly smaller in response to stretch compared to the number of differentially expressed genes in response to 3i-1262 in hiPSC-CMs. Thus, only 15.1% of upregulated and 19.7% of downregulated genes in response to 3i-1262 were reversed by stretch.

Interestingly, metabolic processes were enriched in genes upregulated in stretched 1262-treated hiPSC-CMs *vs*. unstretched DMSO control-treated hiPSC-CMs. On the other hand, the enriched processes related to downregulated genes (stretched 1262-treated *vs*. unstretched DMSO control) did not include processes related to the cell cycle and cell division, whereas processes related to cell adhesion were present. In addition, stretching attenuated the effect of 3i-1262 on key genes associated with maturation (Fig. 6). However, many of the changes, including metabolic maturation-related gene expression, were still significantly upregulated. Interestingly, stretch alone seems to have a similar but smaller (not statistically significant) effect on these genes as 3i-1262. Thus, there is no additive effect when 3i-1262 and stretch are combined, but on the contrary, the effect on maturation-related gene expression is diminished.

## Discussion

Adult mammalian hearts have an inadequate capacity for cardiomyocyte renewal after injury. The discovery of induced pluripotent stem cells and the invention of hiPSC-CMs have revolutionised the fundamentals of human cardiovascular research and therapy [2]. Patient-specific hiPSC-CMs offer several unique benefits and provide an ample cell source for disease modelling, drug research and cell therapies in regenerative medicine. However, to produce appropriate ventricular cells for different cardiovascular applications, the immature cellular status of hiPSC-CMs limits their full potential [3]. hiPSC-CM maturation is an exclusive process—chemical substances driving maturation, such as thyroid hormone and glucocorticoids, fully antagonise the proliferative capacity of cardiomyocytes [3, 9]. Key signalling cascades and intracellular factors regulating cardiomyocyte specification and proliferation are broadly recognised; however, chemical substances controlling cardiomyocyte maturation are still very much undiscovered. Here, we identified novel synthetic small-molecule compounds targeting critical TFs in cardiac development and hypertrophy and explored their ability to change the status of hiPSC-CM maturation.

Several lines of evidence suggest the involvement of GATA4 in the development of cardiac hypertrophy. Our previous results have shown that activation of GATA4, in cooperation with a factor binding to an NKX2-5 binding element, is essential for mechanical stretch-induced cardiomyocyte hypertrophy [18]. Our previous GATA4-NKX2-5 screening campaign produced the first generation of GATA4-acting compounds with cellular activity in a low micromolar range [26, 27]. Our previous results show that modulation of the TF machinery can reduce the hypertrophic growth of cardiac myocytes [26], enhance myocardial repair after myocardial infarction and other cardiac injuries in rodent models [29, 30], and promote atrial and ventricular cell fate [28]. However, to enhance the efficacy and to reduce cell toxicity encountered with our previous lead compound 3i-1000 [30], we synthesised and tested a novel set of structurally optimised compounds. One of these new compounds, 3i-1262, showed improved biological activity on the inhibition of GATA4-NKX2-5 synergy in COS-1 cells, on the BNP promoter activity in NRVMs and on mechanical stretch-induced hypertrophic gene expression in hiPSC-CMs. Moreover, compound 3i-1262 displayed reduced toxicity towards stem cells and no toxicity towards hiPSC-CMs at concentrations up to 30 µM. Structure–toxicity relationship analysis revealed that the observed stem cell toxicity is directly correlated with the size and electrostatics of a substituent attached to the scaffold. Importantly, these results highlight that the efficacy and toxicity of the novel compounds are not perpetually interrelated, providing further options for compound synthesis towards distinct nontoxic structural topologies.

Here, we report that several new compound derivatives, including 3i-1262, inhibit hypertrophic responses in cardiomyocytes. Since ET-1 is a well-established hypertrophic peptide and activates BNP expression, we used an in vitro ET-1 model to investigate the effects of 3i-1262 on cardiomyocyte hypertrophy [26, 35, 50]. In NRVMs, compound 3i-1262, comparable to 3i-1000, inhibited ET-1-induced BNP promoter activity in a concentration-dependent manner without affecting the baseline activity. In addition, both 3i-1000 and 3i-1262, as well as 3i-1047, 3i-1157 and 3i-1183, inhibited ET-1-induced proBNP expression in hiPSC-CMs. Furthermore, compounds 3i-1262 and 3i-1183 inhibited mechanical stretch-induced *NPPB* expression in hiPSC-CMs. Surprisingly, compound 3i-1000 did not inhibit stretch-induced *NPPB* expression in hiPSC-CMs, although we have previously shown that 3i-1000 is capable of inhibiting stretch-induced *Nppb* expression in NRVMs [26], highlighting the difference between cell models from different species. We have also previously reported that the hypertrophic responses and signalling routes are not identical in these two cell models [35, 37]. Overall, these results indicate that compound 3i-1262 is an effective inhibitor of BNP expression, a hallmark of cardiomyocyte hypertrophy, in both NRVMs and hiPSC-CMs.

Our studies further show that compound 3i-1262 consistently decodes cells towards cardiomyocyte identity and maturity. Firstly, 3i-1262 increased cardiomyocyte mitochondrial metabolic activity in the MTT assay even at low concentrations. In addition, RNA analysis of hiPSC-CMs demonstrated that 3i-1262 increases the expression of genes related to several metabolic and biosynthetic processes, as well as key regulators of cardiomyocyte metabolic maturation. Secondly, 3i-1262 downregulated the expression of genes associated with multiple cell cycle and cell division processes. The upregulation of metabolic processes and downregulation of cell cycle processes strongly indicate cardiomyocyte maturation. However, we observed a smaller number of changes in genes related to structural or functional maturation, although increased cTnT expression was observed in stained hiPSC-CMs and several gene expression changes were typical for cardiomyocyte maturation. The modest changes in genes related to structural maturation and the lack of clear maturation response on phenotypic level is a limitation of this study. Apparently, functional maturation requires further quantification with different methods, such as patch clamp and calcium imaging [40]. To attain a comprehensive understanding of the TF network contributing cardiac gene expression, additional studies are needed to elucidate the impact of the ligand on the interplay of the cardiac TFs. Furthermore, investigation of potential off-target effects, exploration of the long-term effects of the GATA4-targeted compound and consideration of in vivo models to validate the observed effects remain interesting topics for further investigation.

## Conclusions

During embryonic development cardiomyocyte fate is established by cardiac TF machinery, and after birth cardiomyocytes predominantly counteract the physiological workload by increasing cell size through hypertrophic signalling. Hypertrophic pathways are initiated by extensive MAPK activation, leading to relocalisation of cardiac TFs towards high-affinity DNA-binding sites, e.g., tandem GATA sites in the BNP promoter, which predominantly halts the developmental signalling pathways linked to cardiomyocyte specification. Persistent activation of hypertrophic pathways is detrimental for cardiomyocytes and leads to heart failure. To this end, cardiac TF machinery is fundamentally devoted to two exclusive processes, myocyte specification and hypertrophy. Systematic understanding regarding the molecular modulation and control of hiPSC-CM maturation is currently inadequate. This study provides the first evidence of the potential of GATA4-targeted compounds in enhancing hiPSC-CM maturation. By using hiPSC-derived cardiomyocyte assays, we developed and optimised our previously published first generation GATA4-acting compounds to yield a safer and more effective chemical modality. Compound-induced perturbation of the TF machinery provides insights into protein‒protein interactions, which have not been conceivable previously. We demonstrated that the GATA4-interfering compound 3i-1262 reorganises the cardiac TF network and converts hypertrophic signalling towards enhanced cardiomyocyte specification and maturity. We assume that parallel compound-induced enhancement of cardiac gene expression and downregulation of hypertrophy markers would benefit patients with hypertrophic cardiomyopathies. Moreover, this finding opens an additional opportunity for the development of hiPSC-CM-based disease models. Hence, this class of compounds can be considered a promising structural scaffold for further development as a cardiomyocyte maturity-promoting modality.

### Supplementary Information

**Additional file 1.** Supplementary methods, tables and figures.

**Additional file 2.** Supplementary table S5 (RNA sequencing).

## Data Availability

The RNAseq data are freely available in the Gene Expression Omnibus (GEO) repository (http://www.ncbi.nlm.nih.gov/geo, accession number GSE186208). All other datasets generated during and analysed during the current study are available from the corresponding author on reasonable request.

## Abbreviations

AURKB: Aurora kinase B (gene)
BrdU: 5-Bromo-2′-deoxyuridin
CACNA1C: Calcium voltage-gated channel subunit alpha1 C (gene)
CCNB1: Cyclin B1 (gene)
CDK1: Cyclin dependent kinase 1 (gene)
cTnT: Cardiac troponin T
DAPI: 4′,6-Diamidino-2-phenylindole
ESRRA: Estrogen related receptor alpha (gene)
ET-1: Endothelin-1
GJA1: Gap junction protein alpha 1/connexin 43 (gene)
GJA5: Gap junction protein alpha 5/connexin 40 (gene)
GO: Gene ontology
HCA: High-content analysis
HCN4: Hyperpolarization activated cyclic nucleotide gated potassium channel 4 (gene)
hiPSC: Human induced pluripotent stem cell
hiPSC-CM: Human induced pluripotent stem cell-derived cardiomyocyte
IGF1R: Insulin like growth factor 1 receptor (gene)
INSR: Insulin receptor (gene)
KCNJ2: Inward rectifier potassium channel 2 (gene)
MAPK: Mitogen-activated protein kinase
MTT: 3-(4,5-Dimethyl-2-thiazolyl)-2,5-diphenyltetrazolium
MYH6: Myosin heavy chain α (gene)
MYH7: Myosin heavy chain β (gene)
NPPA: Atrial natriuretic peptide (gene)
NPPB: B-type natriuretic peptide (gene)
NR3C1: Nuclear receptor subfamily 3 group C member 1 (gene)
NRVM: Neonatal rat ventricular myocyte
PPARA: Peroxisome proliferator-activated receptor alpha (gene)
PPARGC1A: Peroxisome proliferator-activated receptor gamma coactivator 1α (gene)
PPARGC1B: Peroxisome proliferator-activated receptor gamma coactivator 1β (gene)
proBNP: Pro-B-type natriuretic peptide
RNAseq: RNA sequencing
RYR2: Ryanodine receptor 2 (gene)
SCN5A: Sodium voltage-gated channel alpha subunit 5 (gene)
THRA: Thyroid hormone receptor alpha (gene)
THRB: Thyroid hormone receptor beta (gene)
TNNI1: Troponin I1, slow skeletal type (gene)
TNNI3: Troponin I3, cardiac type (gene)
TP53: Tumor protein P53 (gene)
