# Supplementary material for: Switching of hypertrophic signalling towards enhanced cardiomyocyte identity and maturity by a GATA4-targeted compound
Source: Stem Cell Res Ther. 2024 Jan 2;15:5. doi: 10.1186/s13287-023-03623-x (PMC10763434; doi:10.1186/s13287-023-03623-x)
Supplement: Supplementary file 1 — Additional file 1. Supplementary methods, tables and figures. [file 13287_2023_3623_MOESM1_ESM.pdf]

Supplementary information

Switching of hypertrophic signalling towards enhanced cardiomyocyte identity and maturity by a GATA4-targeted compound

Lotta Pohjolainen[1+], Sini M. Kinnunen[1+], Samuli Auno[2], Alexandros Kiriazis[2], Saana Pohjavaara[1], Julia Kari-Koskinen[1], Matej Zore[2], Mikael Jumppanen[2], Jari Yli-Kauhaluoma[2], Virpi Talman[1], Heikki Ruskoaho[1] and Mika J. Välimäki[1*]

[1]*Drug Research Program and Division of Pharmacology and Pharmacotherapy, Faculty of Pharmacy, University of Helsinki, Helsinki, Finland*

[2]*Drug Research Program, Division of Pharmaceutical Chemistry and Technology, Faculty of Pharmacy, University of Helsinki, Helsinki, Finland*

[+]Authors contributed equally to this work

[*]Corresponding author: Mika J. Välimäki, Division of Pharmacology and Pharmacotherapy, Faculty of Pharmacy, University of Helsinki, P.O. Box 56, FI-00014 Helsinki, Finland
email: mika.valimaki@helsinki.fi

Table of Contents

Supplementary Methods	pp. 2–16
Supplementary Tables S1–S4	pp. 17–22
Supplementary Figures S1–S8	pp. 23–30
References	p. 31

Supplementary Methods

1. Synthesis and characterization of compounds

1.1 General procedures

All reactions were carried out using commercially available starting materials acquired from Sigma-Aldrich (Schnelldorf, Germany), Fluka (Buchs, Switzerland), Alfa Aesar (Ward Hill, Massachusetts, USA) and Aurum Pharmatech (Plano, Texas, USA), and were used without further purification. Anhydrous solvents were purchased from Sigma-Aldrich. 3-Cyclopropyl-5-methyl-1,2-oxazole-4-carboxylic acid **4h**, 3-cyclohexyl-5-methyl-1,2-oxazole-4-carboxylic acid **4i**, and 3-cyclopentyl-5-methyl-1,2-oxazole-4-carboxylic acid **4j** were acquired from Enamine (Kyiv, Ukraine). Column chromatography was performed with Merck 230−400 mesh silica gel or with an automated Biotage Isolera Spectra One (Uppsala, Sweden) using a 0.1-mm path length flow cell UV-detector/recorder module (fixed wavelength: 254 nm). Analytical thin layer chromatography (TLC) was carried out using 0.2-mm silica gel plates (silica gel 60, F_{254}, Merck KGaA, Darmstadt, Germany). Microwave reactions were carried out in sealed reaction vessels using a Biotage Initiator+ SP Wave Microwave Synthesizer equipped with an external IR sensor to detect the reaction temperature. Nuclear magnetic resonance spectra (^{1}H NMR at 400 MHz and ^{13}C NMR at 101 MHz) were recorded on Bruker Ascent 400 (Bruker corporation, Billerica, Massachusetts, USA). For $CDCl_3$, CD_2Cl_2, acetone-d_6, MeOH-d_4, DMF-d_7 and DMSO-d_6 the chemical shifts are reported in parts per million (ppm) and on the δ scale using TMS as an internal reference. The coupling constants *J* are quoted in hertz (Hz). Data for ^{1}H NMR spectra are reported as follows: chemical shift (multiplicity, integration, coupling constant(s)). The multiplicity is abbreviated as follows: br = broad signal, s = singlet, d = doublet, t = triplet, q = quartet, dd = doublet of doublets, m = multiplet, m_c = centered multiplet. High resolution mass spectra (HRMS) were measured on a Waters Synapt G2 (Waters Corporation, Milford, Massachusetts, USA) and reported for the molecular ions $[M+H]^+$ or $[M–H]^-$. LC-MS purity analyses were executed with Waters Acquity® UPLC system (Waters, Milford MA, USA) attached to Acquity PDA detector and Waters Synapt G2 HDMS mass spectrometer via an ESI ion source.

Scheme A: Synthesis of target compounds 5a-j (3i-1249 – 3i-1259)

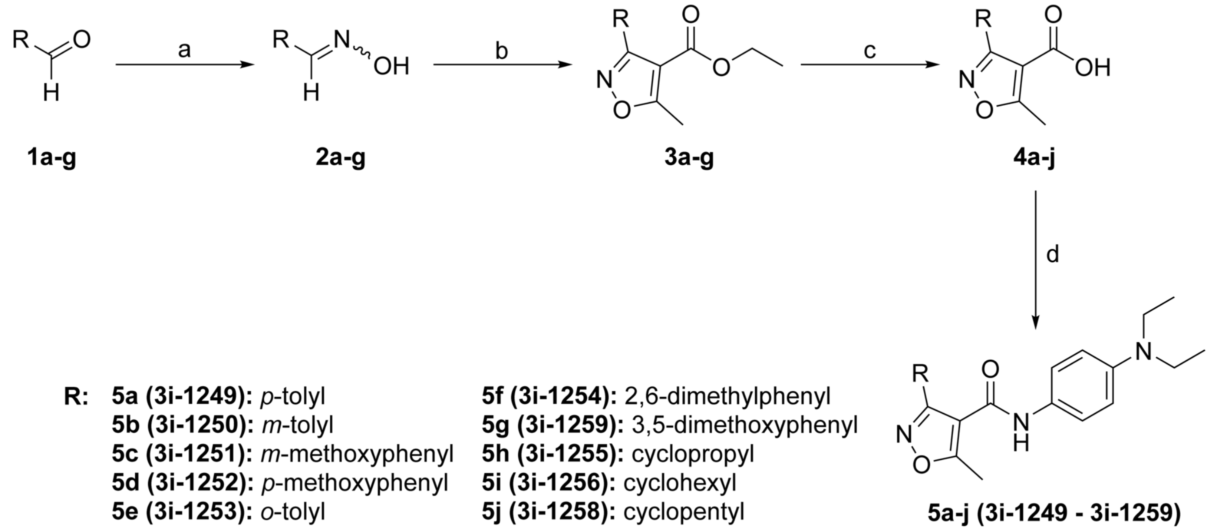

Reagents and conditions: a) $HONH_2$-HCl, pyridine, EtOH, rt, 1-5 h; b) ethyl 2-butynoate, DIB, 2:1 MeCN/water, 0 °C, 40–60 min; c) LiOH, 1:1:1 THF/MeOH/water, rt, 1–3 d; d) *N*,*N*-diethyl-1,4-phenylenediamine, HBTU, DIPEA, DMF, rt, 16-20 h.

Scheme B: Synthesis of target compounds 7-9 and 12 (3i-1260 – 3i-1263)

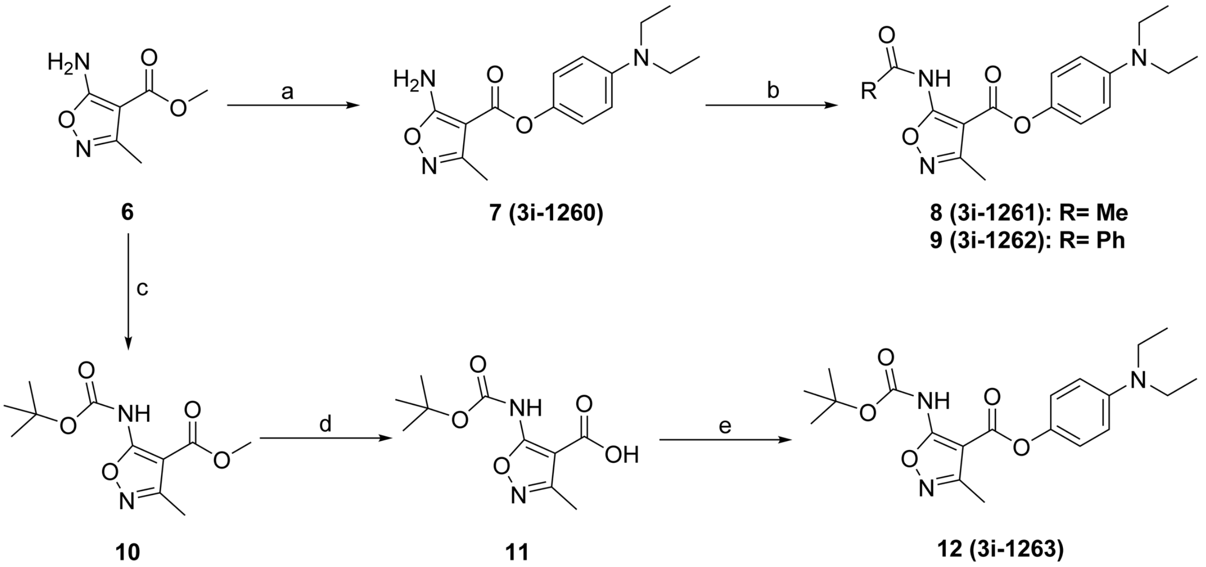

Reagents and conditions: a) DABAL-Me_3, *N*,*N*-diethyl-1,4-phenylenediamine, THF, 70 °C, 21h; b) acetyl chloride (for **8**) or benzoyl chloride (for **9**), 4-DMAP, Et_3N, DCM, rt, 2-3 d; c) Boc_2O, 4-DMAP, DCM, 0 °C, 5 h; d) NaOH (aq), MeOH/H_2O, rt, 5 d; e) *N*,*N*-diethyl-1,4-phenylenediamine, HATU, DIPEA, DMF, rt, 21 h.

General procedure I: Synthesis of aldoximes **2a-g**

Aldehyde **1a-g**, hydroxylammonium chloride (1.1 equiv) and pyridine (1.1 equiv) were dissolved in anhydrous ethanol and stirred at room temperature for 1-5 h. The reaction was quenched with a saturated solution of NH_4Cl in water and the resulting mixture was extracted with dichloromethane (DCM) or ethyl acetate (EtOAc). Combined organic layers were dried over anhydrous sodium sulfate (Na_2SO_4), filtered, and concentrated under reduced pressure. The crude product was purified by flash chromatography on silica gel cartridges using increasing gradient of EtOAc in *n*-heptane as indicated.

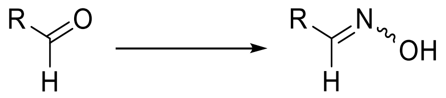

General procedure II: Synthesis of isoxazoles **3a-g** via 1,3-dipolar cycloaddition

Aldoxime **2a-g** and ethyl 2-butynoate (1.5 equiv) were dissolved in a 2:1 mixture of acetonitrile (MeCN) and water at 0 °C. (Diacetoxyiodo)benzene (DIB, 1.2 equiv) was dissolved into equal volume of a 2:1 mixture of MeCN and water, and added dropwise to the reaction mixture during 5 min. The reaction mixture was stirred for 40–120 min, after which time the organic solvent was evaporated under reduced pressure. The aqueous phase was washed with DCM and the resulting organic phase was concentrated under reduced pressure. The crude oil was purified with flash chromatography on silica gel cartridges using increasing gradient of EtOAc in *n*-heptane as indicated.

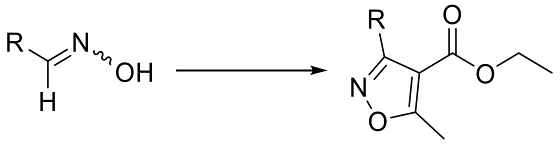

General procedure III: Synthesis of carboxylic acids **4a-g** via ester hydrolysis

Carboxylic acid ester **3a-g** was dissolved in equal volumes of THF, MeOH and water at room temperature. Lithium hydroxide (LiOH, 1.5-2.0 equiv) was added and the mixture was stirred at room temperature for 1-3 days. The mixture was diluted with water and a 1 M solution of NaOH in water, and the aqueous phase was washed with EtOAc or DCM, followed by the addition of EtOAc. The aqueous phase was acidified with a 1 M solution of HCl in water, and the organic phase was washed with water and concentrated under reduced pressure. Resulting carboxylic acids **4a-g** were used in the next step without further purification.

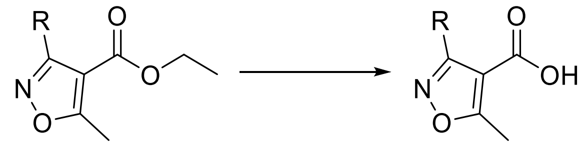

General procedure IV: Synthesis of **5a-j** (**3i-1249 - 3i-1259**) via HBTU-mediated amide coupling

Carboxylic acid **4a-j**, *N,N,N′,N′*-tetramethyl-*O*-(1*H*-benzotriazol-1-yl)uronium hexafluorophosphate (HBTU, 1.3 equiv), *N,N*-diisopropylethylamine (DIPEA, 2.0 equiv) and *N,N*-diethyl-1,4-phenylenediamine (1.0-1.3 equiv) were dissolved in anhydrous *N,N*-dimethylformamide (DMF) under argon at room temperature and the solution was stirred for 16-20 h. Diethyl ether or EtOAc was added, the organic phase was washed with water and concentrated under reduced pressure. The crude product was purified with flash chromatography on silica gel cartridges using increasing gradient of EtOAc in *n*-heptane as indicated.

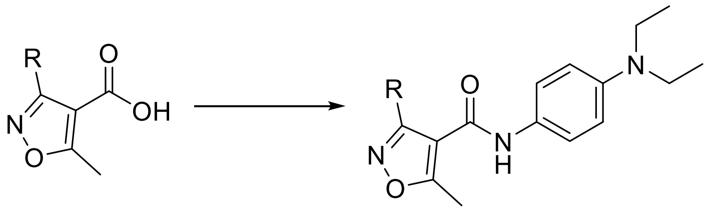

1.2 Synthesis of oximes 2a-g

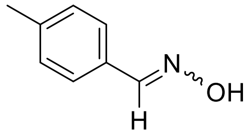

***p*-Tolualdehyde oxime 2a.** Synthesis according to the General procedure I. *p*-Tolualdehyde **1a** (0.500 g, 4.16 mmol), hydroxylammonium chloride (0.318 g, 4.58 mmol), pyridine (0.337 mL, 4.58 mmol), ethanol (10 mL). Reaction time: 4 h. Flash chromatography (8–58% EtOAc in *n*-heptane) gave **2a** as white crystals (76 mg, 13%). ^{1}H NMR (400 MHz, $CDCl_3$) δ 8.13 (s, 1H), 7.50–7.44 (m, 2H), 7.22–7.16 (m, 2H), 2.37 (s, 3H). ^{13}C NMR (101 MHz, $CDCl_3$) δ 150.4, 140.3, 129.5, 129.2 (d, J = 3.8 Hz), 127.0, 21.5.

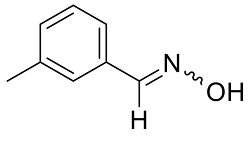

***m*-Tolualdehyde oxime 2b**. Synthesis according to the General procedure I. *m*-Tolualdehyde **1b** (0.500 g, 4.16 mmol), hydroxylammonium chloride (0.318 g, 4.58 mmol), pyridine (0.337 mL, 4.58 mmol), ethanol (10 mL). Reaction time: 2 h. Flash chromatography (8–54% EtOAc in *n*-heptane) gave **2b** as clear, colorless liquid (0.46 g, 82%). ^{1}H NMR (400 MHz, $CDCl_3$) δ 8.15 (s, 1H), 7.43 – 7.37 (m, 2H), 7.29 (t, J = 7.6 Hz, 1H), 7.24 – 7.19 (m, 1H), 2.38 (s, 3H). ^{13}C NMR (101 MHz, $CDCl_3$) δ 150.6, 138.7, 131.9, 131.1, 128.8, 127.7, 124.4, 21.4.

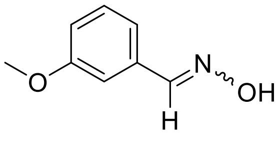

***m*-Anisaldehyde oxime 2c**. Synthesis according to the General procedure I. *m*-Anisaldehyde **1c** (0.500 g, 3.67 mmol), hydroxylammonium chloride (0.281 g, 4.04 mmol), pyridine (0.327 mL, 4.04 mmol), ethanol (10 mL). Reaction time: 2 h. Flash chromatography (0–30% EtOAc in *n*-heptane) gave

2c as a clear, colorless liquid (0.56 g) with residual solvent remaining. ^{1}H NMR (400 MHz, $CDCl_3$) δ 8.13 (s, 1H), 7.30 (t, J = 7.9 Hz, 1H), 7.19 – 7.10 (m, 2H), 6.95 (ddd, J = 8.3, 2.6, 1.0 Hz, 1H), 3.83 (s, 3H). ^{13}C NMR (101 MHz, $CDCl_3$) δ 159.8, 150.4, 133.3, 129.8, 120.1, 116.4, 111.2, 55.3.

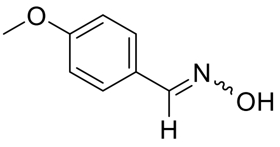

***p*-Anisaldehyde oxime 2d.** Synthesis according to the General procedure I. *p*-Anisaldehyde **1d** (0.500 g, 3.67 mmol), hydroxylammonium chloride (0.281 g, 4.04 mmol), pyridine (0.327 mL, 4.04 mmol), ethanol (10 mL). Reaction time: 3 h. Flash chromatography (5–40% EtOAc in *n*-heptane) gave **2d** as white crystals (0.54 g, 97%). ^{1}H NMR (400 MHz, $CDCl_3$) δ 8.09 (s, 1H), 7.52 (dd, J = 8.9, 2.0 Hz, 2H), 6.91 (dd, J = 8.9, 2.1 Hz, 2H), 3.84 (s, 3H). ^{13}C NMR (101 MHz, $CDCl_3$) δ 161.1, 150.1, 128.5, 124.6, 114.2, 55.4.

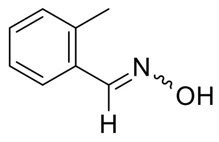

***o*-Tolualdehyde oxime 2e.** Synthesis according to the General procedure I. *o*-Tolualdehyde **1e** (0.500 g, 4.16 mmol), hydroxylammonium chloride (0.318 g, 4.58 mmol), pyridine (0.337 mL, 4.58 mmol), ethanol (10 mL). Reaction time: 5.5 h. Flash chromatography (2–20% EtOAc in *n*-heptane) gave **2e** as a clear, light tan oil (0.43 g, 76%). ^{1}H NMR (400 MHz, $CDCl_3$) δ 8.78 (s, 1H), 8.43 (s, 1H), 7.66 (dd, J = 7.7, 1.5 Hz, 1H), 7.28 (dd, J = 7.4, 1.4 Hz, 1H), 7.23–7.14 (m, 2H), 2.43 (s, 3H). ^{13}C NMR (101 MHz, $CDCl_3$) δ 149.3, 136.8, 130.9, 130.2, 129.9, 126.7, 126.3, 19.8.

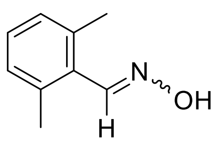

2,6-Dimethylbenzaldehyde oxime 2f. Synthesis according to the General procedure I. 2,6-Dimethylbenzaldehyde **1f** (0.500 g, 3.73 mmol), hydroxylammonium chloride (0.285 g, 4.10 mmol), pyridine (0.332 mL, 4.10 mmol), ethanol (10 mL). Reaction time 5.5 h. Flash chromatography (2–20% EtOAc in *n*-heptane) gave **2f** as white crystals (0.39 g, 71%). ^{1}H NMR (400 MHz, $CDCl_3$) δ 8.44 (s, 1H), 8.36 (s, 1H), 7.17 (dd, J = 8.3, 6.8 Hz, 1H), 7.10–7.05 (m, 2H), 2.42 (s, 6H). ^{13}C NMR (101 MHz, $CDCl_3$) δ 149.8, 137.5, 129.3, 129.0, 128.5, 21.1.

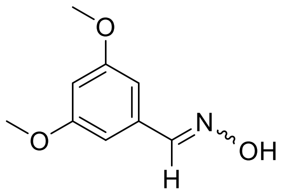

3,5-Dimethoxybenzaldehyde oxime 2g. Synthesis according to the General procedure I. 3,5-Dimethoxybenzaldehyde **1g** (0.500 g, 3.01 mmol), hydroxylammonium chloride (0.230 g, 3.31 mmol), pyridine (0.268 mL, 3.31 mmol), ethanol (10 mL). Reaction time: 2 h. Flash chromatography (10–30% EtOAc in *n*-heptane) gave **2g** as white crystals (0.15 g, 27%). ^{1}H NMR (400 MHz, $CDCl_3$) δ 8.07 (s, 1H), 7.96 (s, 1H), 6.73 (d, J = 2.3 Hz, 2H) 6.50 (t, J = 2.3 Hz, 1H), 3.81 (s, 6H). ^{13}C NMR (101 MHz, $CDCl_3$) δ 161.0, 150.4, 133.8, 104.9, 102.6, 55.5.

1.3 Synthesis of ethyl isoxazole carboxylates 3a-g

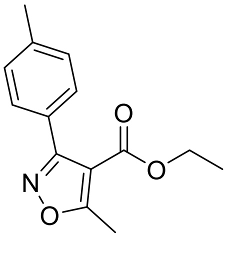

Ethyl 5-methyl-3-(*p*-tolyl)isoxazole-4-carboxylate 3a. Synthesis according to the General procedure II. *p*-Tolualdehyde oxime **2a** (75.5 mg, 0.559 mmol), ethyl 2-butynoate (0.098 mL, 0.838 mmol), DIB (0.216 g, 0.670 mmol), 2:1 $MeCN/H_2O$ (12 mL). Reaction time 38 min. Flash chromatography (2–20% EtOAc in *n*-heptane) gave **3a** as clear, colorless syrup (35 mg, 26%). ^{1}H NMR (400 MHz, $CDCl_3$) δ 7.55 – 7.48 (m, 2H), 7.27 – 7.20 (m, 2H), 4.24 (q, J = 7.1 Hz, 2H), 2.72 (s, 3H), 2.40 (s, 3H), 1.24 (t, J =

7.1 Hz, 3H).^{13}C NMR (101 MHz, $CDCl_3$) δ 175.8, 162.7, 162.2, 139.9, 129.4, 128.8, 125.6, 108.5, 60.8, 31.0, 21.5, 14.2.

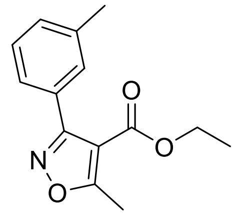

Ethyl 5-methyl-3-(*m*-tolyl)isoxazole-4-carboxylate 3b. Synthesis according to the General procedure II. *m*-Tolualdehyde oxime **2b** (0.198 g, 1.47 mmol), ethyl 2-butynoate (0.256 mL, 2.20 mmol), DIB (0.566 g, 1.76 mmol), 2:1 MeCN/H_2O (20 mL). Reaction time: 40 min. Flash chromatography (5–40% EtOAc in *n*-heptane) gave **3b** as a clear, colorless syrup (70 mg, 19%). ^{1}H NMR (400 MHz, $CDCl_3$) δ 7.46 – 7.39 (m, 2H), 7.37 – 7.27 (m, 2H), 4.24 (q, *J* = 7.1 Hz, 2H), 2.73 (s, 3H), 2.40 (s, 3H), 1.23 (t, *J* = 7.1 Hz, 3H). ^{13}C NMR (101 MHz, $CDCl_3$) δ 175.9, 162.8, 162.1, 137.7, 130.6, 130.0, 128.0, 126.7, 124.6, 108.6, 60.8, 21.5, 14.1, 13.7.

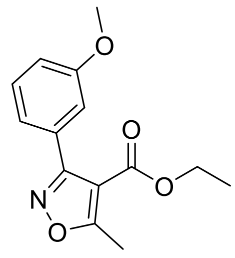

Ethyl 3-(*m*-methoxyphenyl)-5-methylisoxazole-4-carboxylate 3c. Synthesis according to the General procedure II. *m*-Anisaldehyde oxime **2c** (0.200 g, 1.32 mmol), ethyl 2-butynoate (0.231 mL, 1.99 mmol), DIB (0.511 g, 1.59 mmol), 2:1 MeCN/H_2O (20 mL). Reaction time: 1.5 h. Flash chromatography (0–10% EtOAc in *n*-heptane) gave **3c** as a clear, colorless syrup (110 mg, 32%). ^{1}H NMR (400 MHz, $CDCl_3$) δ 7.41–7.30 (m, 1H), 7.23–7.16 (m, 2H), 7.12–6.97 (m, 1H), 4.24 (q, *J* = 7.1 Hz, 2H), 3.83 (s, 3H), 2.73 (s, 3H), 1.23 (t, *J* = 7.1 Hz, 3H). ^{13}C NMR (101 MHz, $CDCl_3$) δ 175.9, 162.5, 162.1, 159.3, 129.8, 129.1, 122.0, 115.8, 114.9, 108.7, 60.8, 55.5, 14.1, 13.7.

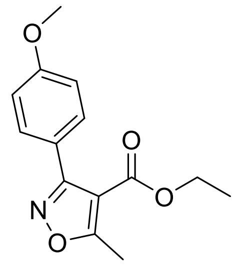

Ethyl 3-(*p*-methoxyphenyl)-5-methylisoxazole-4-carboxylate 3d. Synthesis according to the General procedure II. *p*-Anisaldehyde oxime **2d** (0.200 g, 1.32 mmol), ethyl 2-butynoate (0.231 mL, 1.99 mmol), DIB (0.511 g, 1.59 mmol), 2:1 MeCN/H_2O (20 mL). Reaction time: 2 h. Flash chromatography (0–20% EtOAc in *n*-heptane) gave **3d** as a clear, colorless syrup (86 mg, 25%). ^{1}H NMR (400 MHz, $CDCl_3$) δ 7.63 – 7.56 (m, 2H), 6.99 – 6.93 (m, 2H), 4.26 (q, *J* = 7.1 Hz, 2H), 3.85 (s, 3H), 2.71 (s, 3H), 1.26 (t, *J* = 7.1 Hz, 3H). ^{13}C NMR (101 MHz, $CDCl_3$) δ 190.8, 175.7, 162.2, 162.1, 160.8, 130.8, 120.7, 114.3, 113.5, 108.3, 60.7, 55.3, 14.1, 13.7.

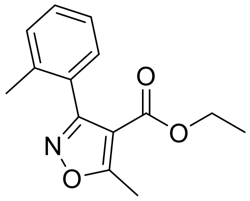

Ethyl 5-methyl-3-(*o*-tolyl)isoxazole-4-carboxylate 3e. Synthesis according to the General procedure II. *o*-Tolualdehyde oxime **2e** (0.200 g, 1.48 mmol), ethyl 2-butynoate (0.259 mL, 2.22 mmol), DIB (0.572 g, 1.78 mmol), 2:1 MeCN/H_2O (20 mL). Reaction time: 1 h. Flash chromatography (0–10% EtOAc in *n*-heptane) gave **3e** as a clear, colorless syrup (184 mg, 51%). ^{1}H NMR (400 MHz, $CDCl_3$) δ 7.37–7.31 (m, 1H), 7.28–7.21 (m, 3H), 4.11 (q, *J* = 7.1 Hz, 2H), 2.76 (s, 3H), 2.20 (d, *J* = 0.7 Hz, 3H), 1.06 (t, *J* = 7.2 Hz, 3H). ^{13}C NMR (101 MHz, $CDCl_3$) δ 175.4, 162.7, 161.9, 137.2, 129.9, 129.6, 129.5, 128.8, 125.4, 109.59, 60.6, 19.9, 13.8, 13.5.

Ethyl 3-(2,6-dimethylphenyl)-5-methylisoxazole-4-carboxylate 3f. Synthesis according to the General procedure II. 2,6-Dimethylbenzaldehyde oxime **2f** (0.200 g, 1,34 mmol), ethyl 2-butynoate (0.234 mL, 2.01 mmol), DIB (0.518 g, 1.61 mmol), 2:1 MeCN/H_2O (20 mL). Reaction time: 50 min. Flash chromatography (0–10% EtOAc in *n*-heptane) gave **3f** as a clear, colorless syrup (255 mg, 73%). ^1H NMR (400 MHz, $CDCl_3$) δ 7.21 (dd, J = 8.1, 7.1 Hz, 1H), 7.09 – 7.05 (m, 2H), 4.07 (q, J = 7.1 Hz, 2H), 2.78 (s, 3H), 2.09 (d, J = 0.8 Hz, 6H), 0.99 (t, J = 7.1 Hz, 3H). ^{13}C NMR (101 MHz, $CDCl_3$) δ 175.7, 161.9, 137.1, 129.0, 128.7, 127.1, 109.3, 60.4, 20.1, 13.7, 13.5.

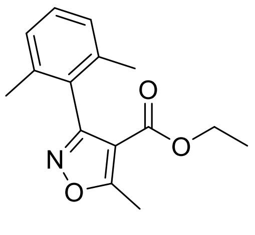

Ethyl 3-(3,5-dimethoxyphenyl)-5-methylisoxazole-4-carboxylate 3g. Synthesis according to the General procedure II. 3,5-Dimethoxybenzaldehyde oxime **2g** (0.100 g, 0.552 mmol), ethyl 2-butynoate (0.096 mL, 0.83 mmol), DIB (0.213 g, 0.662 mmol), 2:1 MeCN/H_2O (10 mL). Reaction time: 1.3 h. Flash chromatography (5–15% EtOAc in *n*-heptane) gave **3g** as a clear, colorless syrup (21 mg, 13%). ^1H NMR (400 MHz, $CDCl_3$) δ 6.78 (d, J = 2.3 Hz, 2H), 6.56 (t, J = 2.3 Hz, 1H), 4.25 (q, J = 7.1 Hz, 2H), 3.81 (s, 6H), 2.72 (s, 3H), 1.24 (t, J = 7.1 Hz, 3H). ^{13}C NMR (101 MHz, $CDCl_3$) δ 175.7, 162.4, 161.9, 160.3, 130.1, 108.6, 107.5, 105.0, 102.1, 60.7, 55.4, 14.0, 13.5.

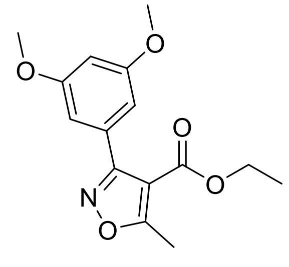

1.4 Synthesis of isoxazolecarboxylic acids 4a-g

5-Methyl-3-(*p*-tolyl)isoxazole-4-carboxylic acid 4a. Synthesis according to the General procedure III. Ethyl 5-methyl-3-(*p*-tolyl)isoxazole-4-carboxylate **3a** (37.9 mg, 0.155 mmol), LiOH (5.6 mg, 0.232 mmol), 1:1:1 THF/MeOH/H_2O (2.4 mL). Reaction time: 24 h. Crude yield: white crystals (21 mg).

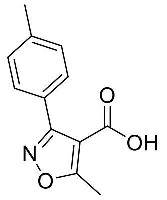

5-Methyl-3-(*m*-tolyl)isoxazole-4-carboxylic acid 4b. Synthesis according to the General procedure III. Ethyl 5-methyl-3-(*m*-tolyl)isoxazole-4-carboxylate **3b** (77.2 mg, 0.315 mmol), LiOH (11.3 mg, 0.472 mmol), 1:1:1 THF/MeOH/H_2O (4.6 mL). An additional portion of LiOH (4.0 mg, 0.17 mmol) was added after 46 h. Reaction time: 50 h. Crude yield: white crystals (56 mg).

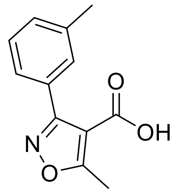

3-(*m*-Methoxyphenyl)-5-methylisoxazole-4-carboxylic acid 4c. Synthesis according to the General procedure III. Ethyl 3-(*m*-methoxyphenyl)-5-methylisoxazole-4-carboxylate **3c** (0.110 g, 0.423 mmol), LiOH (15.2 mg, 0.634 mmol), 1:1:1 THF/MeOH/H_2O (6,6 mL). An additional portion of LiOH (5.0 mg, 0.21 mmol) was added after 23 h. Reaction time: 71 h. Crude yield: white crystals (98 mg).

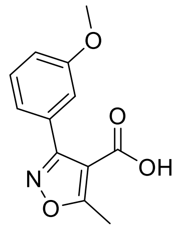

3-(*p*-Methoxyphenyl)-5-methylisoxazole-4-carboxylic acid 4d. Synthesis according to the General procedure III. Ethyl 3-(*p*-methoxyphenyl)-5-methylisoxazole-4-carboxylate **3d** (86.1 mg, 0.330 mmol), LiOH (15.8 mg, 0.657 mmol), 1:1:1 THF/MeOH/H_2O (5.4 mL). Reaction time: 64 h. Crude yield: white crystals (76 mg).

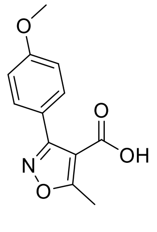

5-Methyl-3-(*o*-tolyl)isoxazole-4-carboxylic acid 4e. Synthesis according to the General procedure III. Ethyl 5-methyl-3-(*o*-tolyl)isoxazole-4-carboxylate **3e** (0.184 g, 0.751 mmol), LiOH (36.0 mg, 1.50 mmol), 1:1:1 THF/MeOH/ H_2O (10.8 mL). Reaction time: 93 h. Crude yield: white crystals (0.15 g).

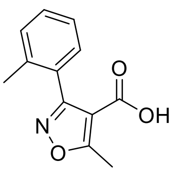

3-(2,6-Dimethylphenyl)-5-methylisoxazole-4-carboxylic acid 4f. Synthesis according to the General procedure III. Ethyl 3-(2,6-dimethylphenyl)-5-methylisoxazole-4-carboxylate **3f** (0.236 g, 0.912 mmol), LiOH (43.7 mg, 1.82 mmol), 1:1:1 THF/MeOH/ H_2O (14 mL). Reaction time: 68 h. Crude yield: white crystals (0.15 g).

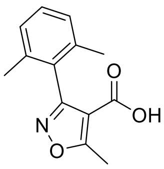

3-(3,5-Dimethoxyphenyl)-5-methylisoxazole-4-carboxylic acid 4g. Synthesis according to the General procedure III. Ethyl 3-(3,5-dimethoxyphenyl)-5-methylisoxazole-4-carboxylate **3g** (63.8 mg, 0.219 mmol), LiOH (10.5 mg, 0.438 mmol), 1:1:1 THF/MeOH/ H_2O (3.9 mL) Reaction time: 65 h. Crude yield: white crystals (54 mg).

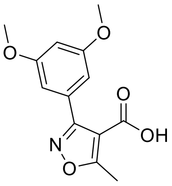

1.5 Synthesis of target compounds 5a-j (3i-1249 - 3i-1259)

***N*-[4-(Diethylamino)phenyl]-5-methyl-3-(*p*-tolyl)isoxazole-4-carboxamide 5a (3i-1249).** Synthesis according to the General procedure IV. 5-Methyl-3-(*p*-tolyl)isoxazole-4-carboxylic acid **4a** (20.9 mg, 0.103 mmol), HBTU (50.7 mg, 0.134 mmol), DIPEA (0.036 mL, 0.206 mmol), *N,N*-diethyl-1,4-phenylenediamine (0.017 mL, 0.103 mmol), DMF (3 mL). Reaction time: 17 h. Flash chromatography (5–50% EtOAc in *n*-heptane) gave **5a** as a light-brown solid (31 mg, 84%). ^{1}H NMR (400 MHz, $CDCl_3$): δ 7.58 – 7.53 (m, 2H), 7.39 – 7.33 (m, 2H), 7.12 – 7.05 (m, 2H), 6.95 (s, 1H), 6.62 – 6.55 (m, 2H), 3.30 (q, *J* = 7.1 Hz, 4H), 2.77 (s, 3H), 2.45 (s, 3H), 1.12 (t, *J* = 7.0 Hz, 6H). ^{13}C NMR (101 MHz, $CDCl_3$) δ 174.6, 159.9, 159.2, 145.4, 140.9, 130.0, 129.1, 125.7, 125.1, 122.1, 112.1, 111.3, 44.5, 21.5, 13.1, 12.5. HRMS-ESI (*m/z*): calcd for $C_{22}H_{25}N_3O_2$ $[M+H]^+$: 364.2025, found 364.2029.

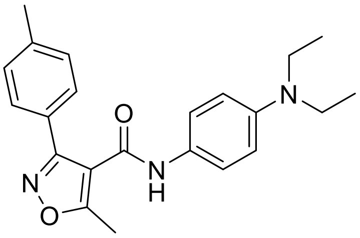

***N*-[4-(Diethylamino)phenyl]-5-methyl-3-(*m*-tolyl)isoxazole-4-carboxamide 5b (3i-1250).** Synthesis according to the General procedure IV. 5-Methyl-3-(*m*-tolyl)isoxazole-4-carboxylic acid **4b** (55.7 mg, 0.274 mmol), HBTU (0.135 g, 0.356 mmol), DIPEA (0.095 mL, 0.548 mmol), *N,N*-diethyl-1,4-phenylenediamine (0.046 mL, 0.274 mmol), DMF (8 mL). Reaction time: 16 h. Flash chromatography (5–30% EtOAc in *n*-heptane) gave **5b** as light-brown solid (50 mg, 50%). ^{1}H NMR (400 MHz, $CDCl_3$) δ 7.51 – 7.49 (m, 1H), 7.48 – 7.45 (m, 2H), 7.43 – 7.38 (m, 1H), 7.14 – 7.06 (m, 2H), 6.99 (s, 1H), 6.64 – 6.57 (m, 2H), 3.33 (q, *J* = 7.1 Hz, 4H), 2.81 (s, 3H), 2.46 (s, 3H), 1.14 (t, *J* = 7.1 Hz, 6H). ^{13}C NMR (101 MHz, $CDCl_3$) δ 174.7, 160.0, 159.1, 145.4, 139.3, 131.4, 129.8, 129.2, 128.1, 126.3, 125.7, 122.0, 112.4, 112.2, 111.3, 44.5, 21.4, 13.1, 12.5. HRMS-ESI (*m/z*): calcd for $C_{22}H_{25}N_3O_2$ $[M+H]^+$: 364.2025, found 364.2029.

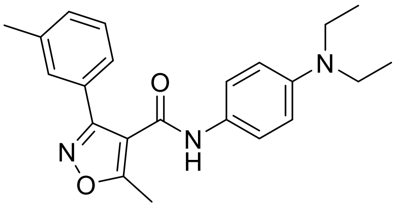

N-[4-(Diethylamino)phenyl]-3-(m-methoxyphenyl)-5-methylisoxazole-4-carboxamide 5c (3i-1251). Synthesis according to the General procedure IV. 3-(*m*-Methoxyphenyl)-5-methylisoxazole-4-carboxylic acid **4c** (98.0 mg, 0.423 mmol), HBTU (0.208 g, 0.547 mmol), DIPEA (0.147 mL, 0.845 mmol), *N,N*-diethyl-1,4-phenylenediamine (0.078 mL, 0.465 mmol), DMF (13 mL). Reaction time: 22 h. Flash chromatography (0–20% EtOAc in *n*-heptane) gave **5c** as a light-brown solid (83 mg, 52%). ^{1}H NMR (400 MHz, $CDCl_3$) δ 7.45 (dd, *J* = 8.4, 7.4 Hz, 1H), 7.24 – 7.20 (m, 1H), 7.18 (dd, *J* = 2.6, 1.5 Hz, 1H), 7.12 – 7.06 (m, 3H), 7.02 (s, 1H), 6.61 – 6.54 (m, 2H), 3.83 (s, 3H), 3.30 (q, *J* = 7.1 Hz, 4H), 2.78 (s, 3H), 1.12 (t, *J* = 7.1 Hz, 6H). ^{13}C NMR (101 MHz, $CDCl_3$) δ 174.8, 160.3, 159.9, 159.2, 145.5, 130.6, 129.5, 125.8, 122.1, 121.4, 117.1, 114.1, 112.3, 111.5, 55.6, 44.6, 13.2, 12.6. HRMS-ESI (*m/z*): calcd for $C_{22}H_{25}N_3O_3$ $[M+H]^+$: 380.1974, found 380.1974.

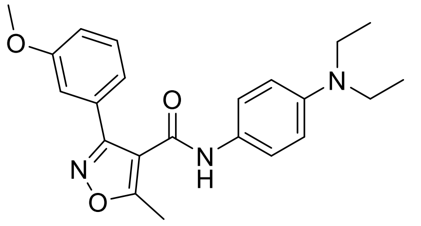

N-[4-(Diethylamino)phenyl]-3-(p-methoxyphenyl)-5-methylisoxazole-4-carboxamide 5d (3i-1252). Synthesis according to the General procedure IV. 3-(*p*-Methoxyphenyl)-5-methylisoxazole-4-carboxylic acid **4d** (76.0 mg, 0.330 mmol), HBTU (0.163 g, 0.428 mmol), DIPEA (0.115 mL, 0.659 mmol), *N,N*-diethyl-1,4-phenylenediamine (0.060 mL, 0.362 mmol), DMF (10 mL). Reaction time: 22 h. Flash chromatography (0–20% EtOAc in *n*-heptane) gave **5d** as a reddish solid (72 mg, 57%). ^{1}H NMR (400 MHz, $CDCl_3$) δ 7.63 – 7.58 (m, 2H), 7.14 – 7.09 (m, 2H), 7.07 – 7.00 (m, 3H), 6.61 – 6.55 (m, 2H), 3.88 (s, 3H), 3.31 (q, *J* = 7.0 Hz, 4H), 2.75 (s, 3H), 1.12 (t, *J* = 7.0 Hz, 6H). ^{13}C NMR (101 MHz, $CDCl_3$) δ 174.5, 161.5, 159.7, 159.4, 145.5, 130.74, 125.8, 122.2, 120.2, 114.9, 112.2, 111.5, 55.6, 44.6, 13.2, 12.6. HRMS-ESI (*m/z*): calcd for $C_{22}H_{25}N_3O_3$ $[M+H]^+$: 380.1974, found 380.1972.

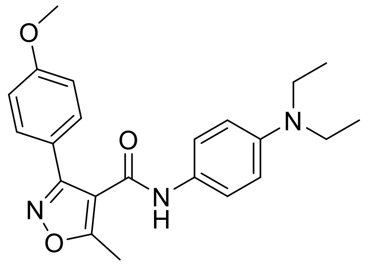

N-[4-(Diethylamino)phenyl]-5-methyl-3-(o-tolyl)isoxazole-4-carboxamide 5e (3i-1253). Synthesis according to the General procedure IV. 5-Methyl-3-(*o*-tolyl)isoxazole-4-carboxylic acid **4e** (0.149 g, 0.684 mmol), HBTU (0.337 g, 0.889 mmol), DIPEA (0.238 mL, 1.37 mmol), *N,N*-diethyl-1,4-phenylenediamine (0.148 mL, 0.889 mmol), DMF (20 mL). Reaction time: 17 h. Flash chromatography (0–20% EtOAc in *n*-heptane) gave **5e** as a yellow syrup (0.22 g, 89%). ^{1}H NMR (400 MHz, $CDCl_3$) δ 7.54 – 7.49 (m, 1H), 7.46 – 7.38 (m, 3H), 6.94 – 6.88 (m, 2H), 6.78 (s, 1H), 6.55 – 6.50 (m, 2H), 3.28 (q, *J* = 7.1 Hz, 4H), 2.85 (s, 3H), 2.27 (s, 3H), 1.10 (t, *J* = 7.0 Hz, 6H). ^{13}C NMR (101 MHz, $CDCl_3$) δ 175.3, 159.5, 158.7, 145.2, 138.1, 131.2, 130.9, 130.1, 127.8, 126.8, 125.9, 121.6, 112.1, 111.4, 44.5, 19.7, 13.4, 12.5. HRMS-ESI (*m/z*): calcd for $C_{22}H_{25}N_3O_2$ $[M+H]^+$: 364.2025, found 364.2022.

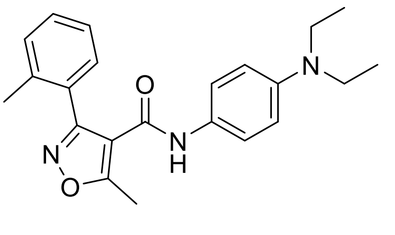

N-[4-(Diethylamino)phenyl]-3-(2,6-dimethylphenyl)-5-methylisoxazole-4-carboxamide 5f (3i-1254). Synthesis according to the General procedure IV. 3-(2,6-Dimethylphenyl)-5-methylisoxazole-4-carboxylic acid **4f** (0.147 g, 0.637 mmol), HBTU (0.314 g, 0.826 mmol), DIPEA (0.222 mL, 1.27 mmol), *N,N*-diethyl-1,4-phenylenediamine (0.138 mL, 0.826 mmol), DMF (20 mL). Reaction time: 23 h. Flash chromatography (0–20% EtOAc in *n*-heptane) gave **5f** as a yellow solid with

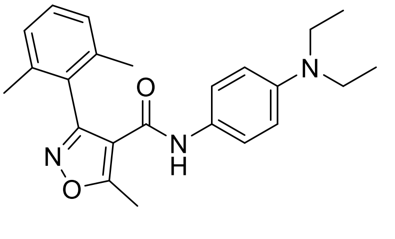

yellow residual oil (0.21 g, 88%). ^{1}H NMR (400 MHz, $CDCl_3$) δ 7.41 (dd, J = 8.1, 7.2 Hz, 1H), 7.29 – 7.23 (m, 2H), 6.90 – 6.85 (m, 2H), 6.82 (s, 1H), 6.54 – 6.49 (m, 2H), 3.27 (q, J = 7.1 Hz, 4H), 2.89 (s, 3H), 2.19 (s, 6H), 1.09 (t, J = 7.1 Hz, 6H). ^{13}C NMR (101 MHz, $CDCl_3$) δ 175.8, 158.6, 158.6, 145.1, 138.3, 130.7, 128.5, 127.4, 126.0, 121.4, 112.1, 110.7, 44.5, 20.0, 13.6, 12.5. HRMS-ESI (*m/z*): calcd for $C_{23}H_{27}N_3O_2$ $[M+H]^+$: 378.2181, found 378.2180.

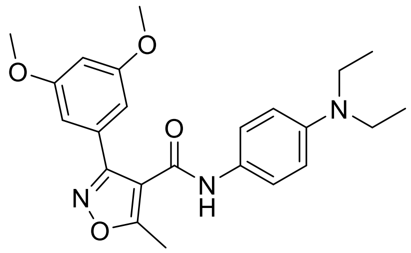

***fN*-[4-(Diethylamino)phenyl]-3-(3,5-dimethoxyphenyl)-5-methylisoxazole-4-carboxamide 5g (3i-1259)**. Synthesis according to the General procedure IV. 3-(3,5-Dimethoxyphenyl)-5-methylisoxazole-4-carboxylic acid **4g** (54.1 mg, 0.206 mmol), HBTU (0.101 g, 0.267 mmol), DIPEA (0.072 mL, 0.411 mmol), *N,N*-diethyl-1,4-phenylenediamine (0.044 mL, 0.267 mmol), DMF (8 mL). Reaction time: 20 h. Flash chromatography (5–20% EtOAc in *n*-heptane) gave **5g** as a light-brown solid (56 mg, 66%). ^{1}H NMR (400 MHz, $CDCl_3$) δ 7.13 (s, 1H), 7.12 – 7.08 (m, 2H), 6.77 (d, J = 2.3 Hz, 2H), 6.63 (t, J = 2.3 Hz, 1H), 6.61 – 6.56 (m, 2H), 3.81 (s, 6H), 3.31 (q, J = 7.1 Hz, 4H), 2.78 (s, 3H), 1.12 (t, J = 7.0 Hz, 6H). ^{13}C NMR (101 MHz, $CDCl_3$) δ 174.8, 161.4, 159.8, 159.0, 145.4, 129.9, 125.7, 122.1, 112.2, 111.3, 106.9, 103.1, 55.6, 44.5, 13.2, 12.5. HRMS-ESI (*m/z*): calcd for $C_{23}H_{27}N_3O_4$ $[M+H]^+$: 410.2080, found 410.2080.

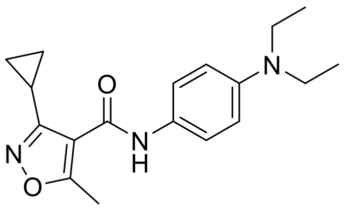

3-Cyclopropyl-*N*-[4-(diethylamino)phenyl]-5-methylisoxazole-4-carboxamide 5h (3i-1255). Synthesis according to the General procedure IV. 3-Cyclopropyl-5-methyl-1,2-oxazole-4-carboxylic acid **4h** (50.0 mg, 0.299 mmol), HBTU (0.147 g, 0.389 mmol), DIPEA (0.104 mL, 0.598 mmol), *N,N*-diethyl-1,4-phenylenediamine (0.065 mL, 0.389 mmol), DMF (9 mL). Reaction time: 18 h. Flash chromatography (0–20% EtOAc in *n*-heptane) gave **5h** as a light-brown solid (76 mg, 68%). ^{1}H NMR (400 MHz, $CDCl_3$) δ 7.90 (s, 1H), 7.41 – 7.34 (m, 2H), 6.71 – 6.63 (m, 2H), 3.34 (q, J = 7.1 Hz, 4H), 2.68 (s, 3H), 2.07 – 1.99 (m, 1H), 1.18 – 1.11 (m, 10H). ^{13}C NMR (101 MHz, $CDCl_3$) δ 174.1, 160.7, 159.8, 145.5, 125.9, 122.3, 112.4, 112.3, 44.6, 13.0, 12.5, 7.1, 6.6. HRMS-ESI (*m/z*): calcd for $C_{18}H_{24}N_3O_2$ $[M+H]^+$: 314.1869, found 314.1868.

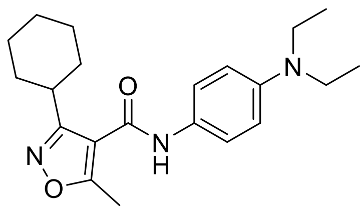

3-Cyclohexyl-*N*-[4-(diethylamino)phenyl]-5-methylisoxazole-4-carboxamide 5i (3i-1256). Synthesis according to the General procedure IV. 3-Cyclohexyl-5-methyl-1,2-oxazole-4-carboxylic acid **4i** (50.0 mg, 0.239 mmol), HBTU (0.118 g, 0.311 mmol), DIPEA (0.083 mL, 0.478 mmol), *N,N*-diethyl-1,4-phenylenediamine (0.052 mL, 0.311 mmol), DMF (8 mL). Reaction time: 21 h. Flash chromatography (0–20% EtOAc in *n*-heptane) gave **5i** as light-brown solid (58 mg, 64%). ^{1}H NMR (400 MHz, $CDCl_3$) δ 7.34 (d, J = 8.3 Hz, 2H), 7.12 (s, 1H), 6.67 (d, J = 8.5 Hz, 2H), 3.35 (q, J = 7.1 Hz, 4H), 3.00 – 2.90 (m, 1H), 2.60 (s, 3H), 2.10 – 1.99 (m, 2H), 1.90 – 1.78 (m, 2H), 1.77 – 1.69 (m, 1H), 1.65 – 1.54 (m, 2H), 1.45 – 1.24 (m, 3H), 1.15 (t, J = 7.0 Hz, 6H). ^{13}C NMR (101 MHz, $CDCl_3$) δ 169.5, 166.0, 145.7, 125.6, 122.6, 112.2, 44.6, 36.2, 31.5, 26.3, 25.9, 12.6 (d, J = 15.3 Hz). HRMS-ESI (*m/z*): calcd for $C_{21}H_{29}N_3O_2$ $[M+H]^+$: 356.2338, found 356.2338.

3-Cyclopentyl-*N*-[4-(diethylamino)phenyl]-5-methylisoxazole-4-carboxamide 5j (3i-1258). Synthesis according to the General procedure IV. 3-Cyclopentyl-5-methyl-1,2-oxazole-4-carboxylic acid **4j** (50.0 mg, 0.256 mmol), HBTU (0.126 g, 0.333 mmol), DIPEA (0.089 mL, 0.512 mmol), *N,N*-diethyl-1,4-phenylenediamine (0.055 mL, 0.333 mmol), DMF (8 mL). Reaction time: 22 h. Flash chromatography (0–30% EtOAc in *n*-heptane) gave **5j** as a light-brown solid (85 mg, 97%). ^{1}H NMR (400 MHz, $CDCl_3$) δ 7.40 – 7.29 (m, 2H), 7.16 (s, 1H), 6.71 – 6.63 (m, 2H), 3.34 (q, J = 7.0 Hz, 4H), 2.60 (s, 3H), 2.16 – 2.04 (m, 2H), 1.97 – 1.85 (m, 2H), 1.85 – 1.75 (m, 2H), 1.72 – 1.62 (m, 2H), 1.15 (t, J = 7.0 Hz, 6H). ^{13}C NMR (101 MHz, $CDCl_3$) δ 170.5, 165.1, 160.4, 145.8, 125.7, 122.7, 112.9, 112.4, 44.7, 37.2, 31.6, 25.5, 12.8, 12.6. HRMS-ESI (*m/z*): calcd for $C_{20}H_{27}N_3O_2$ $[M+H]^+$: 342.2182, found 342.2181.

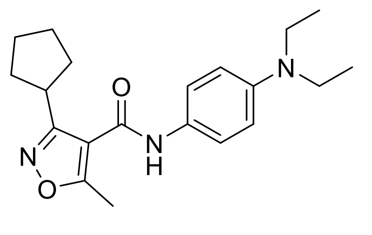

5-Amino-*N*-[4-(diethylamino)phenyl]-3-methylisoxazole-4-carboxamide 7 (3i-1260). Bis(trimethylaluminum)-1,4-diazabicyclo[2.2.2]octane adduct (DABAL-Me_3; 0.246 g, 0.960 mmol) was loaded to a vial and dissolved in anhydrous THF (4 mL) under argon, followed by the addition of *N,N*-diethyl-*p*-phenylenediamine (0.159 mL, 0.956 mmol). Vial was capped and the reaction mixture was heated in oil bath at 70 °C for 1 h. The reaction mixture was let to cool down and methyl 5-amino-3-methylisoxazole-4-carboxylate **6** (0.150 g, 0.961 mmol) in anhydrous THF (5 mL) was added via syringe and the heating was continued at 70 °C for additional 20 h. The reaction mixture was cooled down to 0 °C and quenched carefully by slow addition of water followed by addition of a 1 M solution of HCl in water until no more gas evolved. The acidic aqueous layer was basified with a 1 M solution of NaOH in water and extracted with EtOAc. The combined organic layers were dried over anhydrous Na_2SO_4, filtered, and evaporated under reduced pressure. The crude product was purified by flash chromatography on silica with an increasing gradient of EtOAc in *n*-heptane, to give **7** as a waxy solid (79 mg, 29%). ^{1}H NMR (400 MHz, acetone-d_6) δ 7.65 (br s, 1H), 7.43−7.39 (m, 2H), 7.13 (br s, 2H), 6.69−6.52 (m, 2H), 3.36 (q, J = 6.8 Hz, 4H), 2.48 (s, 3H), 1.12 (t, J = 6.8 Hz, 6H). ^{13}C NMR (101 MHz, $CDCl_3$) δ 172.0, 162.2, 155.8, 145.8, 125.4, 123.7 (2), 112.5 (2), 88.9, 44.7 (2), 12.7, 12.6 (2); LC-MS: $[M+H]^+$, m/z 289 (t_r = 1.08 min), ≥95%; HRMS-ESI (*m/z*): calcd for $C_{15}H_{20}N_4O_2$ $[M+H]^+$: 289.1664; found 289.1663.

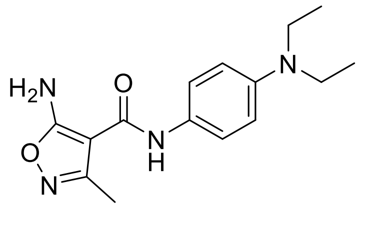

5-Acetamido-*N*-[4-(diethylamino)phenyl]-3-methylisoxazole-4-carboxamide 8 (3i-1261). 5-Amino-*N*-[4-(diethylamino)phenyl]-3-methylisoxazole-4-carboxamide **7** (24.0 mg, 0.083 mmol) and 4-DMAP (16.0 mg, 0.131 mmol, 1.5 equiv) were dissolved in anhydrous DCM (4 mL) followed by addition of triethylamine (115 µL, 0.825 mmol, 10.0 equiv). Then, acetyl chloride (12 µL, 0.168 mmol, 2.0 equiv) was added, and the reaction mixture was stirred at room temperature for 3 d under argon. The reaction mixture was diluted with water and extracted with EtOAc. The combined organic layers were washed with brine, dried over anhydrous Na_2SO_4, filtered, and evaporated under reduced pressure. The crude product was purified by flash chromatography with an increasing gradient of EtOAc in *n*-heptane, to give **8** as a colorless sticky solid (19 mg, 71%). ^{1}H NMR (400 MHz, acetone-d_6) δ 10.32 (br s, 1H), 8.77 (br s, 1H), 7.44 (m_c, 2H), 6.68 (m_c, 2H), 3.37 (q, J = 7.2 Hz, 4H), 2.46 (s, 3H), 2.27 (s, 3H), 1.12 (t, J = 7.2 Hz, 6H). ^{13}C NMR (101 MHz, acetone-d_6): δ 169.2, 162.0, 160.1, 159.7, 146.0,

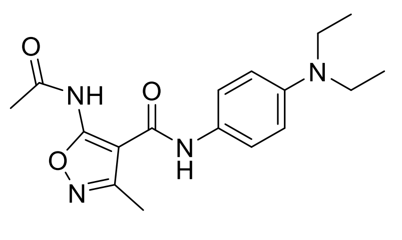

128.1, 122.9 (2), 112.9 (2), 102.1, 45.0 (2), 23.9, 12.9 (2), 11.7; LC-MS: $[M+H]^+$, *m/z* 331 (t_r = 1.50 min), ≥95%; HRMS-ESI (*m/z*): calcd for $C_{17}H_{22}N_4O_3$ $[M+H]^+$: 331.1770; found 331.1771.

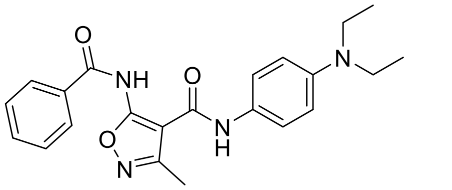

5-Benzamido-*N*-[4-(diethylamino)phenyl]-3-methylisoxazole-4-carboxamide 9 (3i-1262). 5-Amino-*N*-[4-(diethylamino)phenyl]-3-methylisoxazole-4-carboxamide **7** (47.0 mg, 0.163 mmol) and 4-DMAP (31.0 mg, 0.254 mmol, 1.5 equiv) were dissolved in anhydrous DCM (7 mL) followed by addition pyridine (0.140 mL, 1.60 mmol, 10 equiv). Benzoyl chloride (21 µL, 0.181 mmol, 1.1 equiv) was added, and the reaction mixture was stirred at room temperature for 2 d under argon. The reaction mixture was concentrated under reduced pressure, and the crude product was purified by flash chromatography with an increasing gradient of EtOAc in *n*-heptane, to give **9** as a pale-yellow solid (21 mg, 35%). ^{1}H NMR (400 MHz, acetone-d_6) δ 11.36 (br s, 1H), 8.57 (br s, 1H), 8.06–8.03 (m, 2H), 7.69 (m_c, 1H), 7.60 (m_c, 2H), 7.45–7.41 (m, 2H), 6.70–6.65 (m, 2H), 3.37 (q, *J* = 6.8 Hz, 4H), 2.57 (s, 3H), 1.13 (t, *J* = 6.8 Hz, 6H). ^{13}C NMR (101 MHz, acetone-d_6). δ 164.3, 164.2, 160.1, 158.6, 146.2, 133.9, 133.5, 129.9 (2), 128.6 (2), 127.5, 123.7 (2), 112.8 (2), 100.3, 45.0 (2), 12.8 (2), 11.8; LC-MS: $[M+H]^+$, *m/z* 393 (t_r = 2.19 min), ≥95%; HRMS-ESI (*m/z*): calcd for $C_{22}H_{24}N_4O_3$ $[M+H]^+$: 393.1927; found 393.1926. AKI-E85

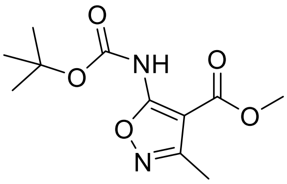

Methyl 5-[(*tert*-butoxycarbonyl)amino]-3-methylisoxazole-4-carboxylate 10. Methyl 5-amino-3-methylisoxazole-4-carboxylate **6** (0.10 g, 0.64 mmol) and 4-DMAP (8.0 mg, 0.060 mmol, 0.1 equiv) were dissolved in DCM (6 mL) and cooled to 0 °C. A solution of di-*tert*-butyl dicarbonate (Boc_2O; 0.15 g, 0.64 mmol) in DCM (3 mL) was added dropwise to the reaction mixture and stirred at 0 °C for 5 h. The reaction mixture was evaporated and purified by flash chromatography on silica with an increasing gradient of EtOAc in *n*-heptane, to give **10** as a white solid (97 mg, 59%). ^{1}H NMR (400 MHz, $CDCl_3$) δ 8.97 (br s, 1H), 3.88 (s, 3H), 2.38 (s, 3H), 1.55 (s, 9 H); ^{13}C NMR (101 MHz, $CDCl_3$) δ 166.3, 164.0, 158.7, 148.8, 91.6, 83.7, 51.9, 28.1 (3), 11.9; LC-MS: [M-*t*-BOC]$^+$, *m/z* 157 (t_r = 3.86 min), ≥95%; HRMS-ESI (*m/z*): calcd for $C_{11}H_{17}N_2O_5$ $[M+H]^+$: 257.1137; found: 257.1135.

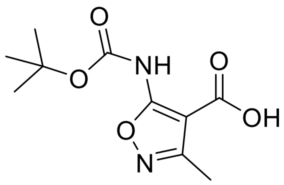

5-[(*tert*-Butoxycarbonyl)amino]-3-methylisoxazole-4-carboxylic acid 11. Methyl 5-[(*tert*-butoxycarbonyl)amino]-3-methylisoxazole-4-carboxylate **10** (97 mg, 0.38 mmol) was dissolved in mixture of MeOH (3 mL) and water (2 mL) followed by addition of a 4 M solution of NaOH in water (3 mL). The resulting mixture was stirred at room temperature for 5 d. The reaction mixture was carefully acidified with a 1 M solution of HCl in water (final pH *ca*. 3) and extracted with chloroform. The combined organic layers were dried over anhydrous Na_2SO_4, filtered, and concentrated to give **11** as a white solid (52 mg, 57%). ^{1}H NMR (400 MHz, DMSO-d_6) δ 13.19 (br s, 1H), 9.77 (br s, 1H), 2.29 (s, 3H), 1.47 (s, 9 H); ^{13}C NMR (101 MHz, Acetone-d_6) δ 167.0, 164.7, 159.7, 149.4, 92.7, 83.4, 28.1 (3), 11.7; LC-MS: [M-*t*-BOC]$^+$, *m/z* (t_r = 2.81 min), ≥95%; HRMS-ESI (*m/z*): calcd for $C_{10}H_{15}N_2O_5$ $[M+H]^+$: 243.0981; found: 243.0978.

***tert*-Butyl [4-[[4-(diethylamino)phenyl]carbamoyl]-3-methylisoxazol-5-yl]carbamate 12 (3i-1263)**. 5-[(*tert*-Butoxycarbonyl)amino]-3-methylisoxazole-4-carboxylic acid **11** (17.0 mg, 0.0702 mmol) and 1-[bis(dimethylamino)methylene]-1*H*-1,2,3-triazolo[4,5-b]pyridinium 3-oxide hexafluorophosphate (HATU; 40.0 mg, 0.105 mmol, 1.5 equiv) were mixed in anhydrous DMF (3 mL) followed by addition of DIPEA (24.0 µL, 0.138 mmol., 2.0 equiv). *N,N*-Diethyl-*p*-phenylenediamine (23.0 µL, 0.138 mmol, 2.0 equiv) was added, and the resulting mixture was stirred at room temperature for 21 h. The reaction mixture was concentrated under reduced pressure, and the crude product was purified by flash chromatography on silica with an increasing gradient of EtOAc in *n*-heptane, to give **12** as an off-white sticky solid (17 mg, 63%). ^{1}H NMR (400 MHz, acetone-d_6) δ 9.79 (br s, 1H), 7.26 (m_c, 2H), 7.12 (br s, 1H), 6.67 (m_c, 2H), 3.35 (q, J = 7.2 Hz, 4H), 2.54 (s, 3H), 1.52 (s, 9H), 1.16 (t, J = 7.2 Hz, 6H); ^{13}C NMR (101 MHz, $CDCl_3$) δ 166.0, 161.4, 155.1, 149.4, 146.1, 124.5, 123.9 (2), 112.3 (2), 93.9, 83.1, 44.7 (2), 28.2 (3), 12.6 (2), 12.5; LC-MS: $[M+H]^+$, m/z 289 (t_r = 2.41 min), ≥95%; HRMS-ESI (m/z): calcd for $C_{10}H_{28}N_4O_4$ $[M+H]^+$: 389.2189; found 389.2188.

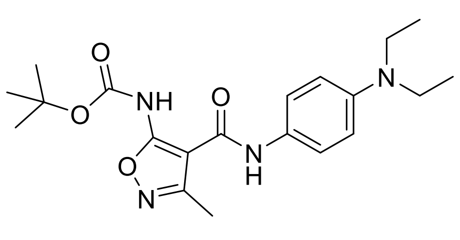

2. AlphaScreen

The AlphaScreen (Amplified Luminescent Proximity Homogenous Assay) method was pre-validated by optimising the following parameters: amount of plasmids in transfections, the lysis buffers, expression levels of tagged proteins and their cross-titrations for optimal signal intensity for GATA4-NKX2-5 interaction. Shortly, COS-1 cells were seeded onto 6-well plates at 300,000 cells per well and transfected next day with 3 µg of pDEST40-GATA4-C-V5 or 2.4 µg of pcDNA™5/FRT/TO-NKX2-5-N-SH using FuGene6 (Promega) in 3:1 ratio to DNA. After 24 h the cells were detached by trypsin and counted with hemocytometer. Medium was removed by centrifugation 200 *g* at 4 °C for 4 min and the cells were washed once with phosphate-buffered saline (PBS). Finally, the cells were suspended into non-denaturing lysis buffer (20 mM Tris-HCl, 150 mM NaCl, 1 mM EDTA, 1 mM EGTA, 1% Triton-X100, 2.5 mM sodium pyrophosphate, pH 7.5) with phosphatase inhibitors (1 mM β-glycerophosphate, 1 mM Na_3VO_4, 50 mM NaF) and protease inhibitors (Protease Inhibitor Mini Tablets, #88666, Pierce) to contain 4,000 cells per µL. The cells were disrupted by vortexing vigorously for 20 s followed by centrifugation at 15 000 *g* for 20 min at 4 °C. The supernatant containing the total proteins was transferred into clean sample tube. GATA4 and NKX2-5 protein lysates were diluted into AlphaScreen sample buffer (50 mM Tris-HCl pH7.4, 150 nM NaCl, 0.1% BSA) added to ½ AreaPlate-96 (#6002290, Perkin Elmer) in addition to dilution series of compound or DMSO into AlphaScreen sample buffer and incubated at 4 °C for 60 min. Each sample well contained proteins from 1,000 cells with GATA4 overexpression and 2,500 cells with NKX2-5 overexpression. V5-acceptor beads (AL129, Perkin Elmer) and Strep-Tactin donor beads (AS106, Perkin Elmer) were added 20 µg/mL in final concentration and incubated at room temperature covered from light for 60 min. The plate was analysed using Enspire Alpha plate reader (Perkin Elmer).

3. Immunofluorescence staining

The cells were washed twice with PBS and fixed with 4% paraformaldehyde at room temperature for 15 min and permeabilized with 0.1% Triton X-100 in PBS at room temperature for 10 min. Non-specific binding sites were blocked with 4% FBS in PBS for 45 min followed by the addition of primary

antibodies diluted in 4% FBS in PBS: cardiac troponin T (cTnT) antibody (Abcam Cat# ab45932) at 1:800, proBNP antibody (Abcam Cat# ab13115) at 1:200, BrdU antibody (Abcam Cat# ab6326) at 1:250, and GATA4 antibody (Cell Signaling Technology Cat# 36966) at 1:400. After a 60-min incubation at room temperature, the cells were washed 3 × 5 min with PBS and incubated with Alexa Fluor®-conjugated secondary antibodies (Life Technologies, Eugene, Oregon) at 1:200, and 4′,6-diamidino-2-phenylindole (DAPI; Sigma-Aldrich) at 1 µg/mL at room temperature for 45 min. The cells were then washed 3 × 5 min with PBS and stored at 4 °C in PBS until imaged.

4. qPCR

For qPCR, 100–500 ng of total RNA was reverse-transcribed in 10 µL reactions using a Transcriptor First Strand cDNA Synthesis Kit (Roche, Basel, Switzerland) according to the manufacturer's protocol using random hexamer primers and an MJ Mini Personal thermal cycler (Bio-Rad, Hercules, CA, USA). The cDNA was diluted 1:10 in PCR grade H2O and stored at -20 °C. The commercial TaqMan® Gene Expression Assays (Thermo Fisher Scientific) for atrial natriuretic peptide (*NPPA*, Hs00383230_g1), B-type natriuretic peptide (*NPPB*, Hs01057466_g1), PPARG coactivator 1 alpha (*PPARGC1A*, Hs00173304_m1), cyclin B1 (*CCNB1*, Hs01030099_m1), aurora kinase B (*AURKB*, Hs00945858_g1), myosin heavy chain 6 (*MYH6*, Hs01101425_m1), myosin heavy chain 7 (*MYH7*, Hs01110632_m1), beta-actin (*ACTB*, 4333762T), and eukaryotic 18S ribosomal RNA (*18S rRNA*, 4352930E) were used with LightCycler® 480 Probes Master reagent (Roche) according to the manufacturer’s instructions. A LightCycler® 480 Real-Time PCR System (Roche) was used to analyse 4.5 µL of the cDNA dilution in 10 µL reactions on a white LightCycler® 480 Multiwell Plate 384 (Roche). No-template controls were used to confirm the absence of PCR contamination. Each reaction was run at least in duplicate, and the average of the technical replicates was used in the analysis as N=1. The 2-ΔΔCt method was used to analyse the relative gene expression using *ACTB* and *18S rRNA* as reference genes.

5. RNA sequencing

RNA sequencing (RNAseq) was performed as single-end sequencing for a read length of 75 bp with an Illumina NextSeq 500 sequencer (Illumina, San Diego, CA, USA) in high output runs using a NEBNext Ultra Directional RNA Library Prep kit (New England Biolabs, Ipswich, MA, USA) including rRNA depletion. Data quality was analysed by FastQC, and quality trimming was applied to the data with Trimmomatic software.[1] The sample reads were aligned against the Genome Reference Consortium Human Build 38 patch release 13 (GRCh38.p13, GCA_000001405.28) reference with Spliced Transcripts Alignment to a Reference (STAR).[2] Mapping quality was assessed with Qualimap.[3] Read quantification was created with featureCounts[4], and differential expression with quality assessment was performed with DESeq2.[5]

6. Western blotting

The effects of endothelin-1 (ET-1; 100 nM) and compound **3i-1262** (30 µM) on α-actinin, cardiac troponin T, and pro-B-type natriuretic peptide (proBNP) protein expression in hiPSC-CMs was investigated with western blotting. hiPSC-CMs were plated on 6-well plates at a density of 2×10^6 cells / well and maintained in RB+ until day 30 of differentiation. The cells were treated with **3i-1262** or vehicle 1 h prior to addition of ET-1. Brefeldin A was added to the cells three hours before cell lysis. After the 24 h exposure, cells were lysed with 1% sodium dodecyl sulphate in 50 mM Tris-HCl (pH 7.5).

To shear the genomic DNA, a 25G needle was used. Protein concentrations of the samples were determined with the Pierce™ BCA Protein Assay -kit (Thermo Scientific). Fifteen micrograms of total protein were loaded on 10% Mini-PROTEAN® TGX Stain-Free™ Protein gels (Bio-Rad) and proteins were separated by electrophoresis. To activate the reaction between trihalo compounds and proteins, which enables the visualisation of total proteins, the gels were exposed to UV-light for 5 min. Next, proteins were transferred to a nitrocellulose membrane by Trans-Blot® Turbo™ transfer system (Bio-Rad). Nonspecific background was blocked with 5% non-fat dry milk in Tris-buffered saline with 0.1 % Tween 20 (TTBS) for 1 h at RT. The membranes were incubated with primary antibody 1:1000 solution (anti α-actinin (Abcam, ab7811), anti-cTnT (Abcam, ab45932) and anti-proBNP (Abcam, ab13115)) in 5% milk-TTBS overnight at 4°C. The membranes were washed with TTBS, followed by a 1-hour incubation in the 1:2000 secondary antibody solutions (anti-mouse (Cell Signaling Technology, 7076); anti-rabbit (Cell Signaling Technology, 7074S)) conjugated with Precision Protein StrepTactin-HRP Conjugate (Bio-Rad). α-actinin and cTnT bands were detected with an enhanced chemiluminescent substrate SuperSignal™ West Pico PLUS (Thermo Scientific) whereas proBNP bands were detected with SuperSignal™ West Femto Maximum Sensitivity Substrate (Thermo Scientific) using a ChemiDoc™ MP Imaging System (Bio-Rad). Optical densities of the bands were quantified using the ImageLab 6.0.1. software (Bio-Rad). The densities of the protein bands were normalised to the total protein of the sample.

Supplementary Tables

Supplementary Table S1. Kinase screening results for compound **3i-1262** at 10 µM concentration (ScanEDGE, Eurofins DiscoveRx, San Diego).

Gene Symbol	Compound 3i-1262 % Ctrl @ 10 µM	Gene Symbol	Compound 3i-1262 %Ctrl @ 10 µM
ABL1 (E255K)-phosp.	94	KIT (D816V)	100
ABL1 (T315I)-phosp.	99	KIT (V559D,T670I)	100
ABL1-nonphosp.	66	LKB1	75
ABL1-phosphor.	95	MAP3K4	96
ACVR1B	80	MAPKAPK2	95
ADCK3	91	MARK3	77
AKT1	100	MEK1	83
AKT2	82	MEK2	100
ALK	66	MET	96
AURKA	94	MKNK1	80
AURKB	71	MKNK2	81
AXL	76	MLK1	91
BMPR2	87	p38-alpha	95
BRAF	98	p38-beta	88
BRAF (V600E)	98	PAK1	100
BTK	80	PAK2	92
CDK11	66	PAK4	100
CDK2	91	PCTK1	98
CDK3	100	PDGFRA	91
CDK7	77	PDGFRB	93
CDK9	90	PDPK1	99
CHEK1	99	PIK3C2B	79
CSF1R	100	PIK3CA	81
CSNK1D	93	PIK3CG	96
CSNK1G2	99	PIM1	100
DCAMKL1	87	PIM2	100
DYRK1B	78	PIM3	100
EGFR	62	PKAC-alpha	100
EGFR (L858R)	100	PLK1	80
EPHA2	100	PLK3	86
ERBB2	75	PLK4	95
ERBB4	84	PRKCE	82
ERK1	98	RAF1	100
FAK	99	RET	81
FGFR2	100	RIOK2	90
FGFR3	89	ROCK2	79
FLT3	76	RSK2	77
GSK3B	100	SNARK	65
IGF1R	97	SRC	100
IKK-alpha	96	SRPK3	85
IKK-beta	92	TGFBR1	90
INSR	95	TIE2	75
JAK2 (JH1domain)	78	TRKA	68
JAK3 (JH1domain)	100	TSSK1B	70
JNK1	64	TYK2 (JH1domain)	78
JNK2	70	ULK2	100
JNK3	70	VEGFR2	100
KIT	100	YANK3	72
		ZAP70	83

Supplementary Table S2. Summary chart of study compounds measured for GATA4-NKX2-5 synergy in luciferase reporter assay and viability in human induced pluripotent stem cells (hiPSC).

Code	Molecule structure	GATA4-NKX2-5 synergy at 10 µM (% of control)	hiPSC viability at 30 µM (% of control)	Supplier	Reference
**3i-1000**	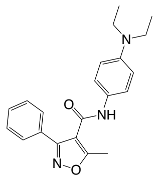	62<br>20[a]<br>47[b]<br>50[c]	10<br>6[b]	Pharmatory Oy<br>Oulu, Finland	This study<br>[a]Välimäki *et al.* 2017<br>[b]Karhu *et al.* 2018<br>[c]Jumppanen *et al.* 2019
**3i-1022**	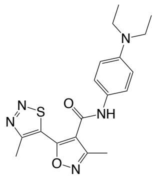	24[c]	–	Faculty of Pharmacy<br>Univ. of Helsinki	[c]Jumppanen *et al.* 2019
**3i-1047**	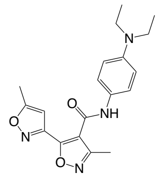	83[b]<br>78[c]	62[b]	Faculty of Pharmacy<br>Univ. of Helsinki	[b]Karhu *et al.* 2018<br>[c]Jumppanen *et al.* 2019
**3i-1157**	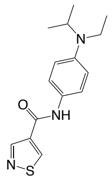	12[c]	69	Faculty of Pharmacy<br>Univ. of Helsinki	[c]Jumppanen *et al.* 2019<br>This study
**3i-1183**	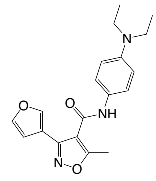	110[c]	11	Faculty of Pharmacy<br>Univ. of Helsinki	[c]Jumppanen *et al.* 2019<br>This study
**3i-1249**	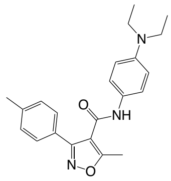	96	82	Faculty of Pharmacy<br>Univ. of Helsinki	This study
**3i-1250**	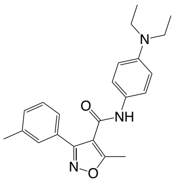	92	9	Faculty of Pharmacy<br>Univ. of Helsinki	This study
**3i-1251**	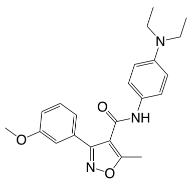	94	64	Faculty of Pharmacy<br>Univ. of Helsinki	This study

**3i-1252**	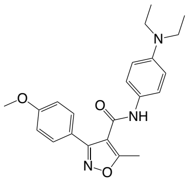	101	51	Faculty of Pharmacy Univ. of Helsinki	This study
**3i-1253**	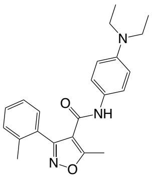	78	9	Faculty of Pharmacy Univ. of Helsinki	This study
**3i-1254**	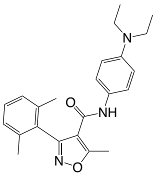	72	22	Faculty of Pharmacy Univ. of Helsinki	This study
**3i-1255**	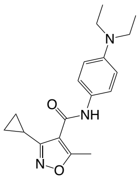	97	102	Faculty of Pharmacy Univ. of Helsinki	This study
**3i-1256**	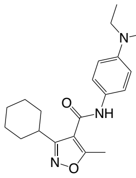	59	16	Faculty of Pharmacy Univ. of Helsinki	This study
**3i-1258**	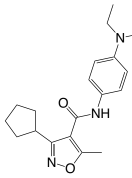	83	17	Faculty of Pharmacy Univ. of Helsinki	This study
**3i-1259**	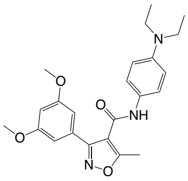	75	47	Faculty of Pharmacy Univ. of Helsinki	This study
**3i-1260**	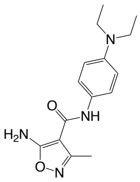	113	67	Faculty of Pharmacy Univ. of Helsinki	This study
**3i-1261**	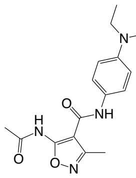	76	67	Faculty of Pharmacy Univ. of Helsinki	This study

**3i-1262**	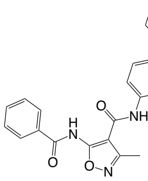	36	31	Faculty of Pharmacy Univ. of Helsinki	This study
**3i-1263**	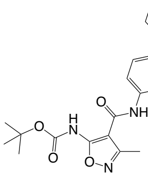	58	57	Faculty of Pharmacy Univ. of Helsinki	This study

Supplementary Table S3. KEGG pathways enriched in upregulated genes in response to **3i-1262** in human pluripotent stem cell-derived cardiomyocytes (hiPSC-CMs).

KEGG identifier	Description	Enrichment ratio	FDR p-value
hsa01230	Biosynthesis of amino acids	4.37	8.96E-09
hsa00100	Steroid biosynthesis	8.08	1.69E-07
hsa01100	Metabolic pathways	1.53	4.34E-07
hsa04216	Ferroptosis	4.97	1.89E-06
hsa01200	Carbon metabolism	2.95	1.25E-05
hsa03320	PPAR signalling pathway	3.49	4.46E-05
hsa00020	Citrate cycle (TCA cycle)	4.85	8.90E-05
hsa01212	Fatty acid metabolism	3.87	1.17E-04
hsa01210	2-Oxocarboxylic acid metabolism	6.06	1.49E-04
hsa04931	Insulin resistance	2.64	5.84E-04
hsa00900	Terpenoid backbone biosynthesis	4.96	9.20E-04
hsa04920	Adipocytokine signalling pathway	3.03	0.00116546
hsa00061	Fatty acid biosynthesis	6.61	0.002484647
hsa00330	Arginine and proline metabolism	3.03	0.009031363
hsa00250	Alanine, aspartate and glutamate metabolism	3.63	0.009989596
hsa04975	Fat digestion and absorption	3.63	0.009989596
hsa00071	Fatty acid degradation	3.19	0.013833441
hsa00620	Pyruvate metabolism	3.19	0.013833441
hsa04911	Insulin secretion	2.39	0.019445435
hsa04211	Longevity regulating pathway	2.31	0.019445435
hsa00630	Glyoxylate and dicarboxylate metabolism	3.46	0.022331563
hsa04142	Lysosome	2.04	0.023514586
hsa04710	Circadian rhythm	3.23	0.033146145
hsa00260	Glycine, serine and threonine metabolism	2.87	0.041850579

Supplementary Table S4. KEGG pathways enriched in downregulated genes in response to **3i-1262** in human pluripotent stem cell-derived cardiomyocytes (hiPSC-CMs).

**KEGG identifier**	**Description**	**Enrichment ratio**	**FDR p-value**
hsa04110	Cell cycle	5.19	1.09E-13
hsa03030	DNA replication	8.42	1.15E-09
hsa03460	Fanconi anemia pathway	5.46	4.08E-06
hsa05166	Human T-cell leukemia virus 1 infection	2.47	2.51E-04
hsa04512	ECM-receptor interaction	3.40	0.003027269
hsa04218	Cellular senescence	2.51	0.006295923
hsa03440	Homologous recombination	4.16	0.010346239
hsa04914	Progesterone-mediated oocyte maturation	2.86	0.020100585
hsa04510	Focal adhesion	2.14	0.020100585
hsa03430	Mismatch repair	4.94	0.031615404
hsa03410	Base excision repair	4.02	0.040113094
hsa04360	Axon guidance	2.09	0.040113094
hsa05226	Gastric cancer	2.26	0.040113094
hsa05212	Pancreatic cancer	2.78	0.040113094
hsa04930	Type II diabetes mellitus	3.44	0.040113094

Supplementary Figures

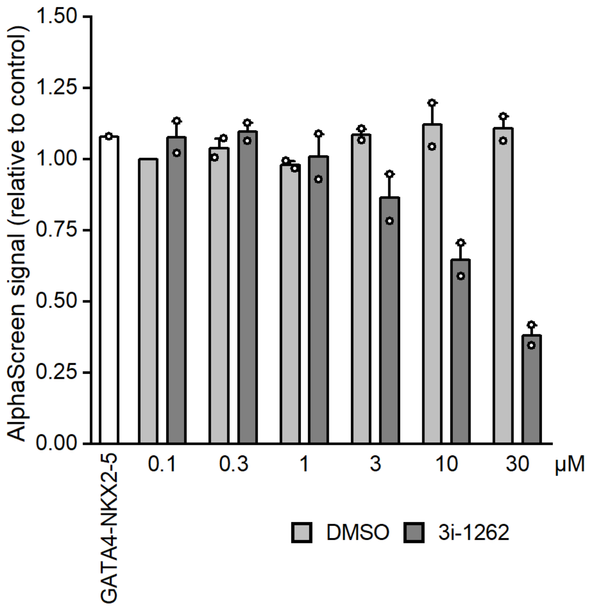

Supplementary Figure S1. Compound **3i-1262** demonstrated concentration-dependent inhibition of GATA4-NKX2-5 interaction measured by bead-based assay technology AlphaScreen. Results are expressed as mean of two independent experiments adjusted to 0.1 µM DMSO control ± STDEV (n=2, GATA4-NKX2-5 n=1).

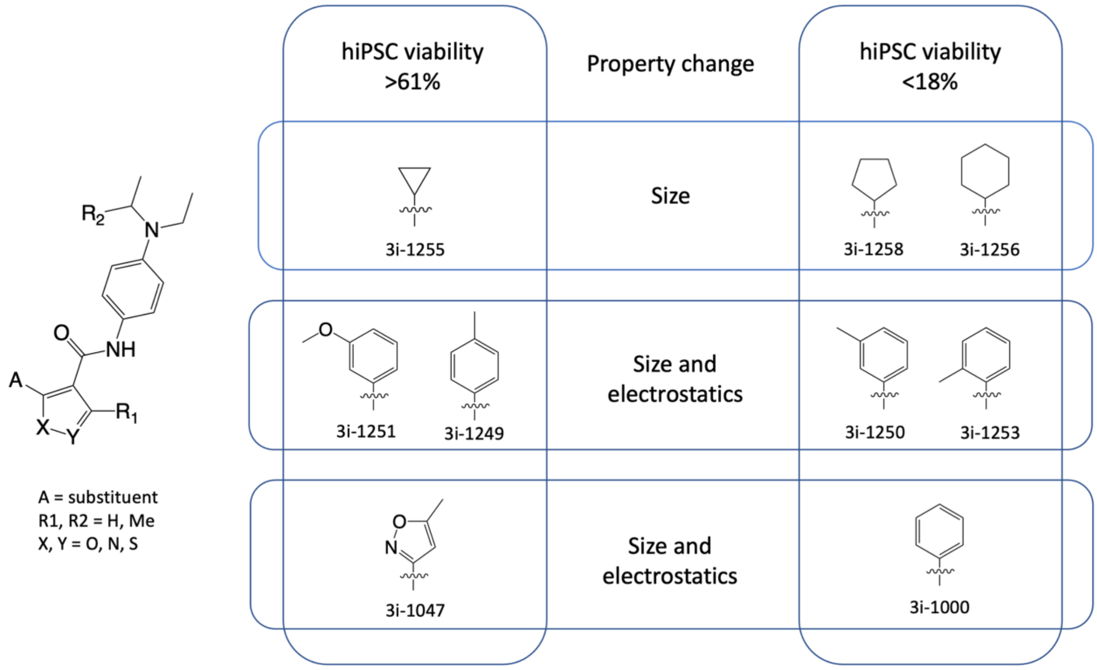

Supplementary Figure S2. Underlying hiPSC toxicity is noticed at 30 µM concentration by various compound derivatives with different substituent size and electrostatic properties. Top row, spatial requirement of the substituent ring associates to the stem cell toxicity (cyclopropyl vs. cyclopentyl and cyclohexyl). Middle row, the electron-donating substituent (methyl and methoxy) in phenyl ring reduces stem cell toxicity if the transverse space demand does not increase (methyl in *ortho*- and *para*-position). Bottom row, simultaneous employment of reduced space requirement (5-membered ring) and electron-donating group (methyl) decreases stem cell toxicity.

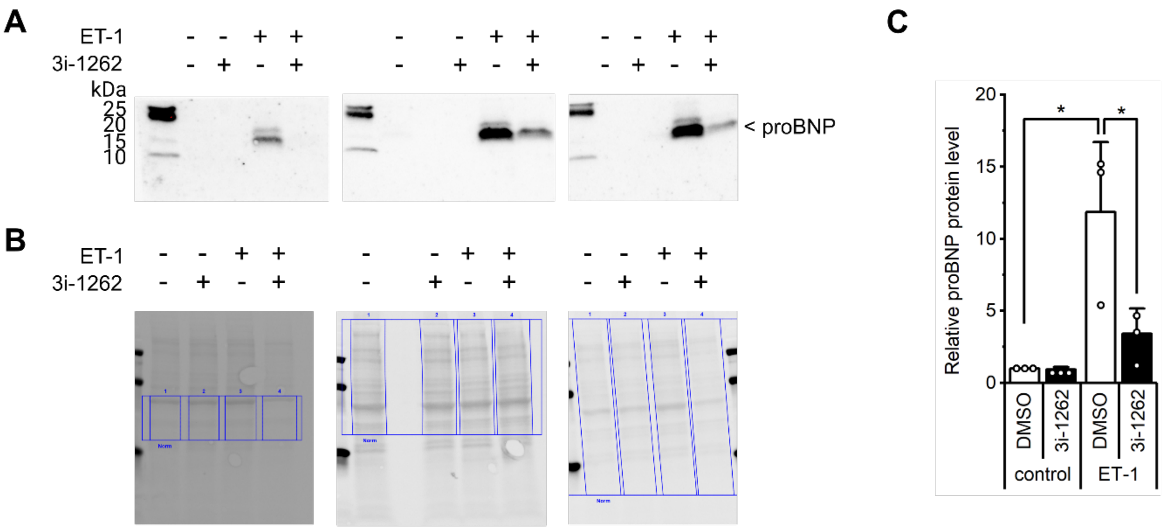

Supplementary Figure S3. Effects of endothelin-1 (ET-1) and compound **3i-1262** on pro-B-type natriuretic peptide (proBNP) protein levels after a 24 h exposure. hiPSC-CMs were exposed to **3i-1262** at 30 µM and ET-1 at 100 nM after which the relative protein levels were determined and normalised to the total protein. (A) Western blots for proBNP. (B) Images of the total protein staining. Quantification areas are shown in blue. (C) Quantification of proBNP expression in hiPSC-CMs. The results are presented as mean ±SEM (N=3), *p<0.05 as indicated (Mann-Whitney U test).

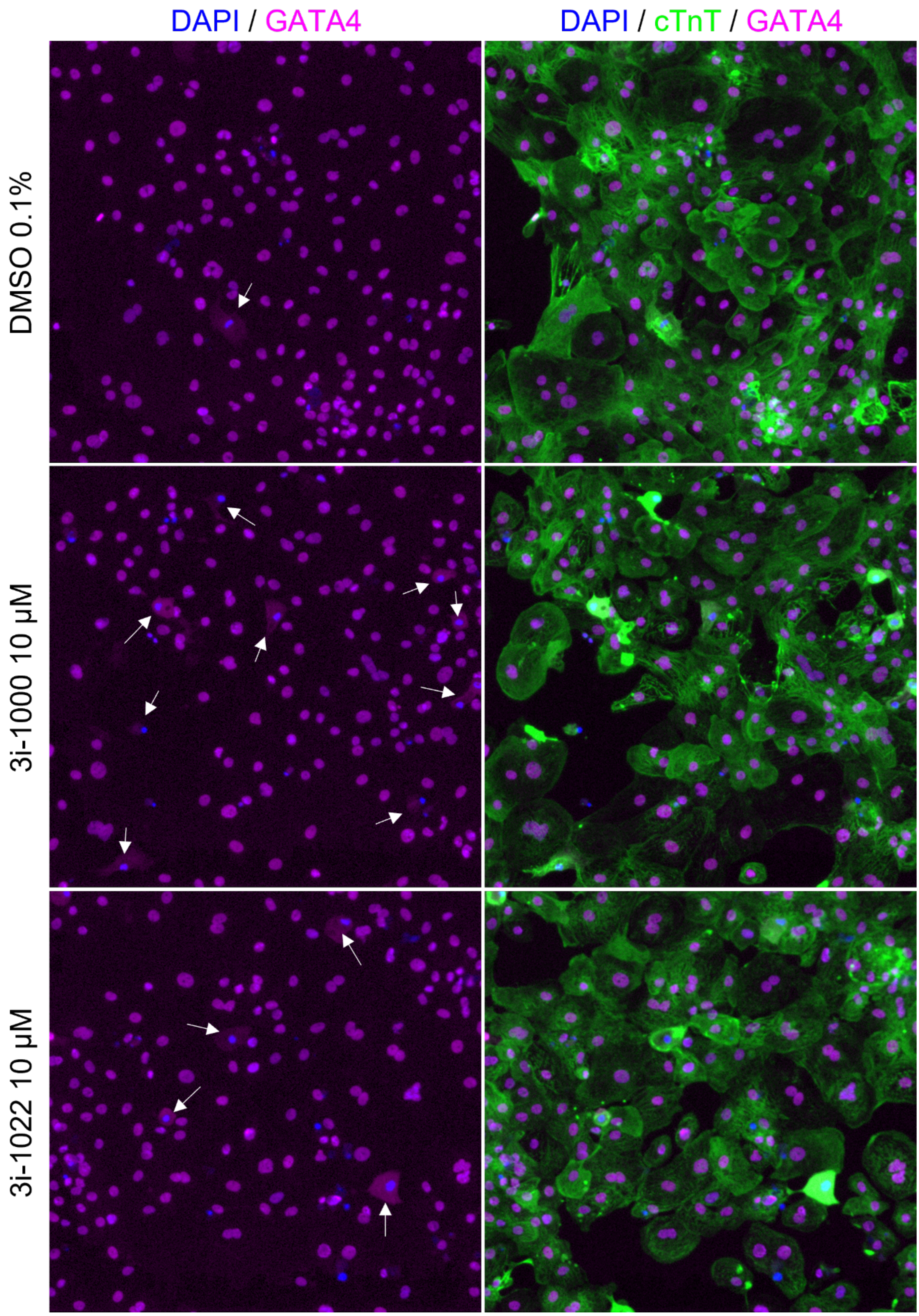

Supplementary Figure S4. Representative images of DAPI, GATA4 and cTnT-stained hiPSC-CMs showing increased cytosolic GATA4 expression in response to compounds **3i-1000** and **3i-1022**.

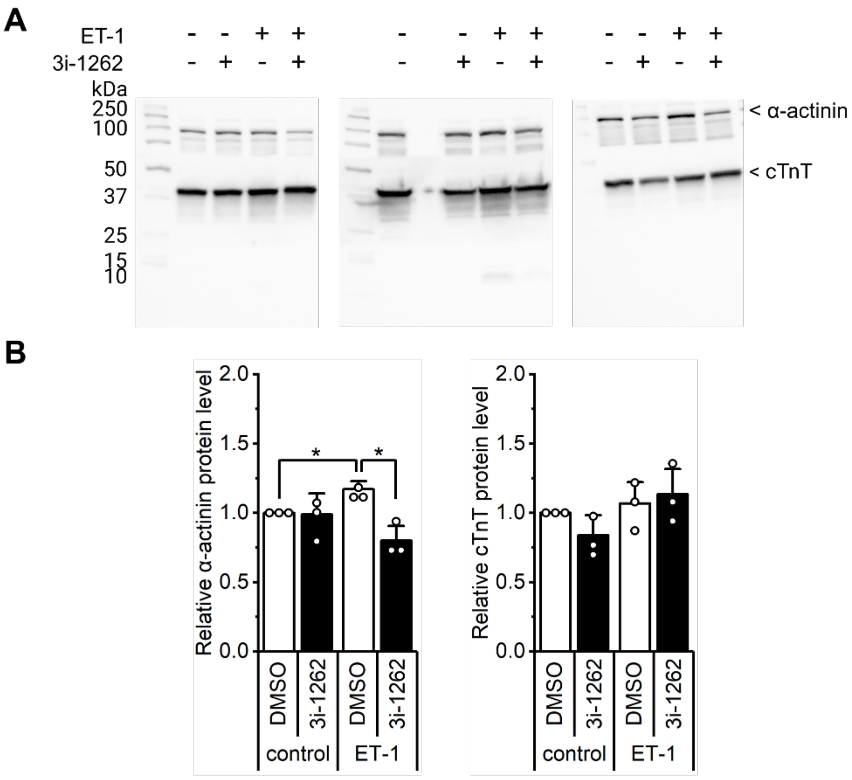

Supplementary Figure S5. Effects of endothelin-1 (ET-1) and compound **3i-1262** on cardiac α-actinin and cardiac troponin T (cTnT) protein levels after a 24 h exposure. hiPSC-CMs were exposed to **3i-1262** at 30 µM and ET-1 at 100 nM after which the relative protein levels were determined and normalised to the total protein. (A) Western blot of α-actinin and cTnT. (B) Quantification of α-actinin and cTnT in hiPSC-CMs. Images of the total protein staining and quantification areas are shown in *Supplementary Figure S3*. The results are presented as mean ±SEM (N=3), *p<0.05 as indicated (Mann-Whitney U test).

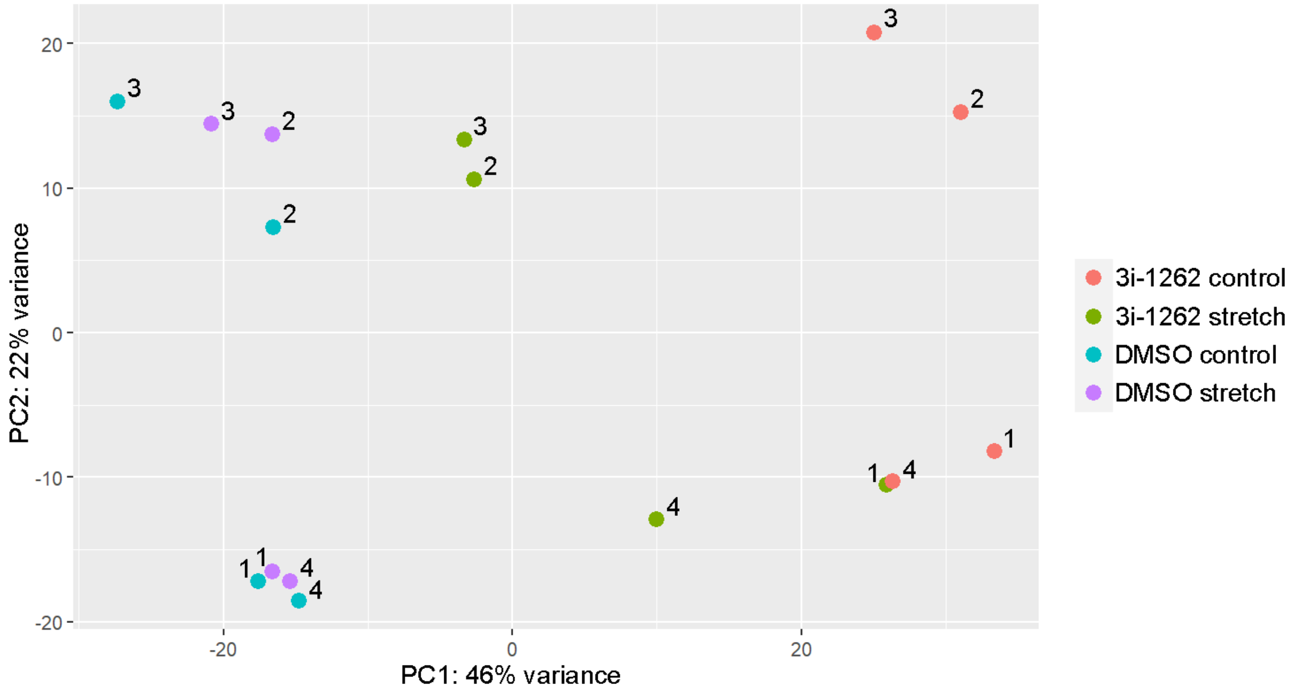

Supplementary Figure S6. Two-dimensional principal component (PC) analysis of the RNA sequencing results of 72 h stretched and unstretched (control) human induced pluripotent stem cell-derived cardiomyocytes (hiPSC-CMs) treated with **3i-1262** or DMSO (vehicle). Each dot indicates a single sample. The samples are numbered based on individual experiments (1–4). The RNAseq results of the stretch response without **3i-1262** (DMSO only) have been published previously.[6]

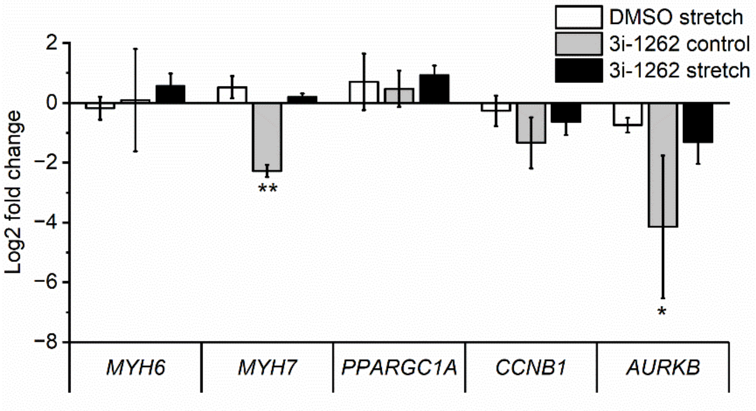

Supplementary Figure S7. Relative mRNA expression changes in hiPSC-CMs were measured by qPCR after a 72 h exposure to compound **3i-1262** at 30 µM and/or cyclic mechanical stretch. Results are presented as log2-fold changes ±SEM (N=3) relative to the unstretched DMSO control. *p<0.05, **p<0.001 vs. the unstretched DMSO control; One-way ANOVA followed by Tukey's post-hoc test or Welch ANOVA followed by Games-Howell.

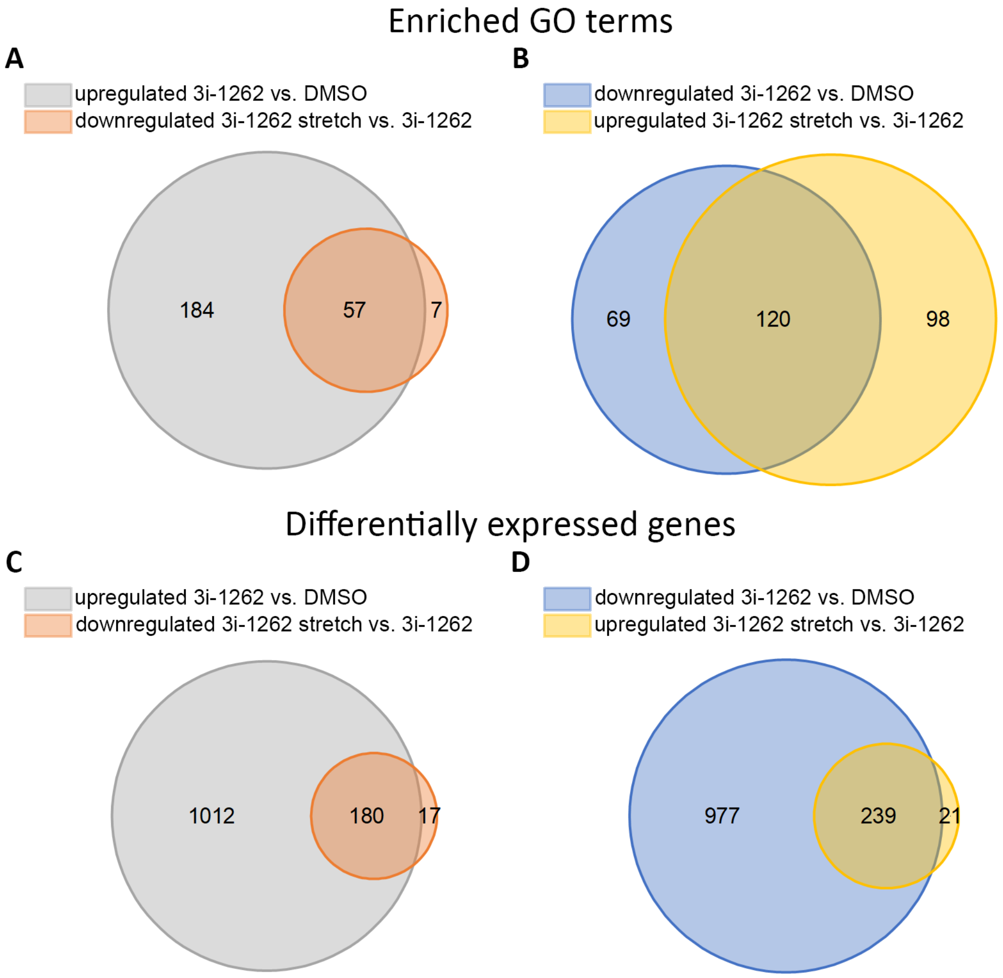

Supplementary Figure S8. Mechanical stretch counteracts the transcriptomic changes of compound **3i-1262**. Venn diagrams show the number of enriched gene ontology (GO) terms (A, B) and differentially expressed genes (C, D) which were dysregulated in response to **3i-1262** vs. DMSO and **3i-1262** stretch vs. **3i-1262**.

References

1. Bolger AM, Lohse M, Usadel B. Trimmomatic: a flexible trimmer for Illumina sequence data. *Bioinformatics* 2014;**30**:2114–2120.
2. Dobin A, Davis CA, Schlesinger F, Drenkow J, Zaleski C, Jha S, Batut P, Chaisson M, Gingeras TR. STAR: ultrafast universal RNA-seq aligner. *Bioinformatics* 2013;**29**:15–21.
3. Okonechnikov K, Conesa A, García-Alcalde F. Qualimap 2: advanced multi-sample quality control for high-throughput sequencing data. *Bioinformatics* 2016;**32**:292–294.
4. Liao Y, Smyth GK, Shi W. featureCounts: an efficient general purpose program for assigning sequence reads to genomic features. *Bioinformatics* 2014;**30**:923–930.
5. Love MI, Huber W, Anders S. Moderated estimation of fold change and dispersion for RNA-seq data with DESeq2. *Genome Biol* 2014;**15**:550.
6. Pohjolainen L, Ruskoaho H, Talman V. Transcriptomics reveal stretched human pluripotent stem cell-derived cardiomyocytes as an advantageous hypertrophy model. *J Mol Cell Cardiol Plus* 2022;**2**:100020.
